# Microtomographic Analysis of a Palaeolithic Wooden Point from the Ljubljanica River †

**DOI:** 10.3390/s22062369

**Published:** 2022-03-18

**Authors:** Enej Guček Puhar, Lidija Korat, Miran Erič, Aleš Jaklič, Franc Solina

**Affiliations:** 1 Computer Vision Laboratory, Faculty of Computer and Information Science, University of Ljubljana, Večna Pot 113, SI-1000 Ljubljana, Slovenia; ales.jaklic@fri.uni-lj.si; 2 The Laboratory for Cements, Mortars and Ceramics, Slovenian National Building and Civil Engineering Institute, Dimičeva Ulica 12, SI-1000 Ljubljana, Slovenia; lidija.korat@zag.si; 3 Institute for the Protection of Cultural Heritage of Slovenia, Poljanska 40, SI-1000 Ljubljana, Slovenia; miran.eric@guest.arnes.si

**Keywords:** computer vision, computed tomography, 3D surface-based models, 3D volumetric models, Palaeolithic wooden point, conservation, waterlogged wood, archaeological documentation, heritage science

## Abstract

A rare and valuable Palaeolithic wooden point, presumably belonging to a hunting weapon, was found in the Ljubljanica River in Slovenia in 2008. In order to prevent complete decay, the waterlogged wooden artefact had to undergo conservation treatment, which usually involves some expected deformations of structure and shape. To investigate these changes, a series of surface-based 3D models of the artefact were created before, during and after the conservation process. Unfortunately, the surface-based 3D models were not sufficient to understand the internal processes inside the wooden artefact (cracks, cavities, fractures). Since some of the surface-based 3D models were taken with a microtomographic scanner, we decided to create a volumetric 3D model from the available 2D tomographic images. In order to have complete control and greater flexibility in creating the volumetric 3D model than is the case with commercial software, we decided to implement our own algorithm. In fact, two algorithms were implemented for the construction of surface-based 3D models and for the construction of volumetric 3D models, using (1) unsegmented 2D images CT and (2) segmented 2D images CT. The results were positive in comparison with commercial software and new information was obtained about the actual state and causes of the deformation of the artefact. Such models could be a valuable aid in the selection of appropriate conservation and restoration methods and techniques in cultural heritage research.

## 1. Introduction

**Figure 1:**
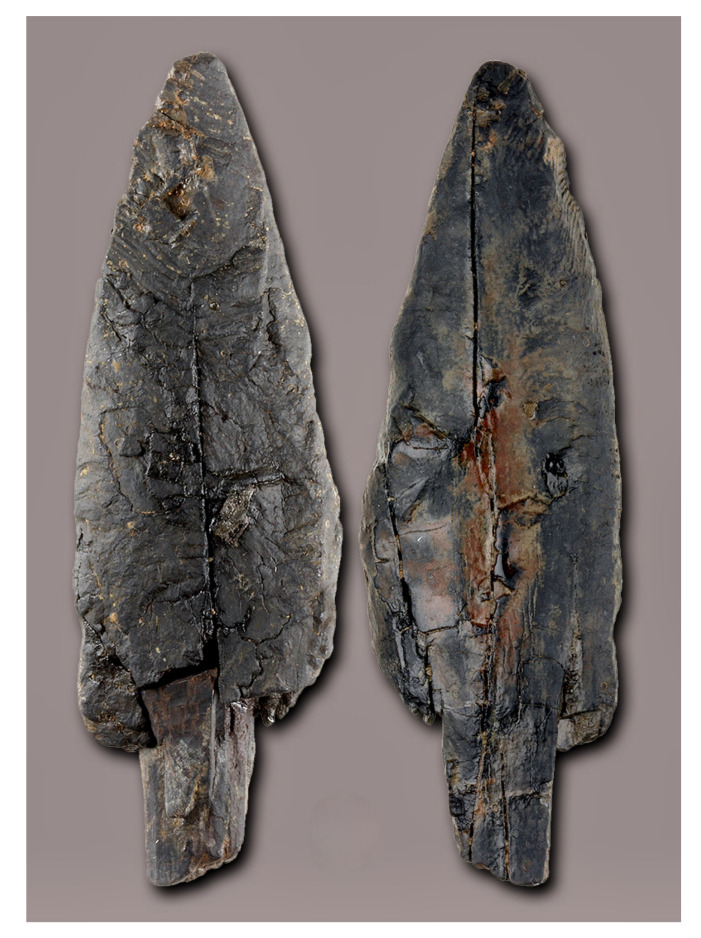
The 40,000 years old Palaeolithic wooden point, only one out of eight wooden artefacts of such age known in Europe, was found in 2008, submerged in the river Ljubljanica at Sinja Gorica [2]. As any waterlogged wooden artefact it had to undergo a conservation process to prevent it’s complete deterioration once taken out of water [4]. The wooden point before conservation (photo by Arhos d.o.o.).

**Figure 2:**
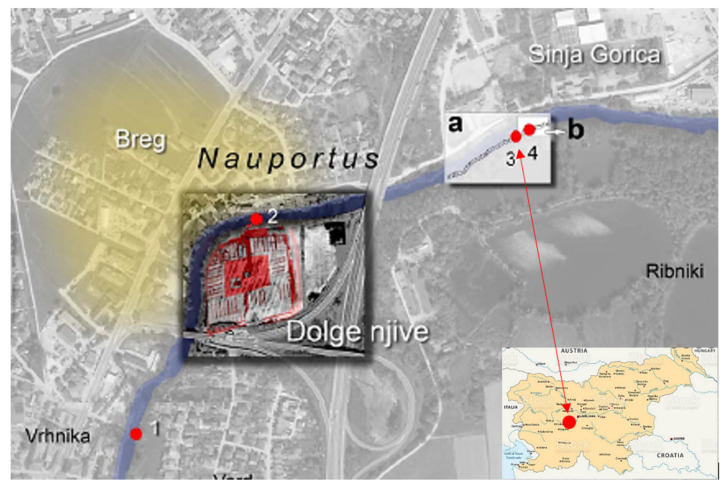
Northeastern area of Vrhnika (Slovenia) with the Nauportus area (a; b) and the location (3) where the Paleolithic wooden point was found in the river Ljubljanica.

Our interest in reconstructing 3D solid models using computed tomography was piqued when we were confronted with the deformation changes of a Palaeolithic wooden point [1] (Figure 1). The 40,000-year-old wooden point was found underwater in the Ljubljanica River close to Sinja Gorica (Figure 2) in 2008 [2]. The Ljubljanica River flows in that part on the Ljubljana Moor, which is dotted with numerous prehistoric pile dwellings, some of which are also on the UNESCO World Heritage List. The entire moor is under the special protection by the state authorities and the wooden point was found during a protection survey in the Ljubljanica River. Near the wooden point a flat-bottomed shipwreck from the Roman period was also found [3].

Additional laboratory investigations of the artefact (age and type of wood; unnatural/manual processing of the wood with other tools or fire) were carried out. Radiometric analysis (AMS14C) places the point in the Palaeolithic period. Two datings have been made. The first (Miami–USA) showed yew wood (Taxus sp.) to be older than 43,970 years (Beta-252943), and a re-dating in Oxford gave a maximum age of 38,490 ± 330 BP (OxA-19866).

Since the discovery, special attention and care have been taken to ensure conditions for the permanent preservation of the point in its natural environment. Underwater archaeologists suggested sinking the point in a special capsule in the deeper sediments of the site in the riverbed of the Ljubljanica. In this way, the point would be optimally protected from the danger of rapid decay. The 3D model and the virtual replica would be presented to the public. However, a different view prevailed. This envisaged the conservation and public presentation of the Palaeolithic remains as a valuable museum exhibit. The lace was kept for five years under artificial conditions (in distilled and regularly changing water). In 2013, the lace was sent to the Römisch–Germanisches Zentralmuseum in Mainz (Germany), which carried out the conservation with melamine resin (C3 H6 N6) in the years 2013–2015. The conservation was completed in 2017. In 2018, the artefact was returned to the Ljubljana City Museum, where it is kept. The wooden point is now one of only eight Palaeolithic wooden artefacts in Europe. However, no other artefact was made in such a beautiful leaf-like shape.

**Figure 3:**
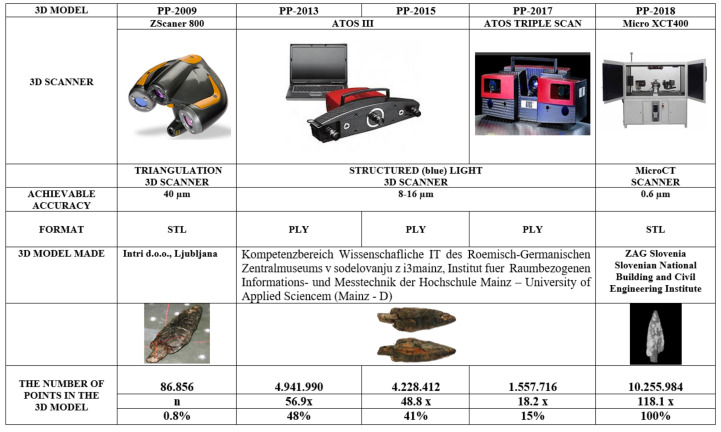
3D scanners used to capture the five 3D models of the palaeolithic wooden point in different years.

A visual inspection of the point after conservation revealed that it had changed. The point was smaller, lighter in colour and more clearly curved in the area of the planting. A computerised volumetric and geometric deformation analysis of the point cloud of five 3D models (CloudCompare computer software) confirmed this finding. Significant deterioration and deformation was found during the conservation phase. In 2018, we conducted a comparison of five 3D surface models of the Palaeolithic wooden point [5]. The artefact was scanned in 2009–2018 using optical scanners and microtomographic scanners, i.e., before, during and after the consevation process (Figure 3). The comparative analysis of the 3D surface models was performed with the Iterative closest point (ICP) algorithm and the open-source software CloudCompare, which showed that the deformation process had slowed down but was still present. These circumstances prompted us to conduct an additional microtomographic examination of the anatomical structure of the artefact. The ability to compare several 3D models of the same object is very important for broader conservation treatment and the cultural heritage community [6].

**Figure 4:**
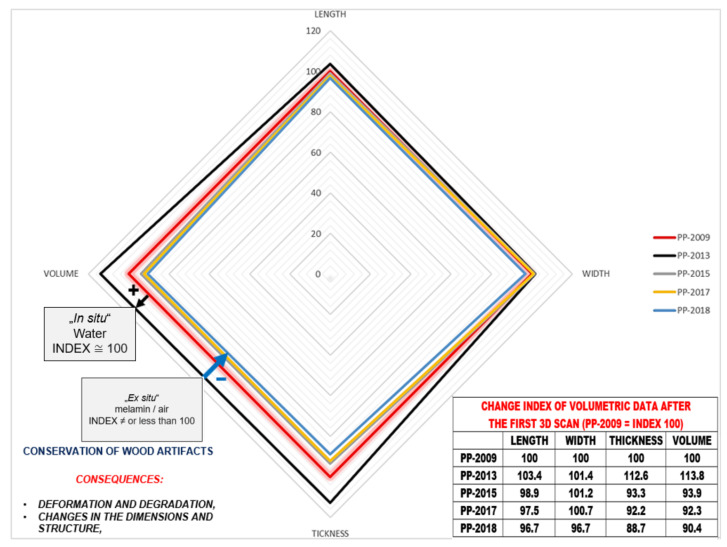
Index of change in volumetric data after the first scan (2009—index 100). The polar diagram shows the dynamics of volumetric changes (length, width, thickness and volume) between five 3D models of the Palaeolithic wooden point recorded between 2009 and 2018. The red line represents the volumetric state of the point at the first 3D scan (2009—index 100), which reflects the approximation of the state of the artefact under In situ conditions. The diagram clearly shows the changes that occurred at the beginning of the conservation process (2013—black line), when the artefact was subjected to intensive soaking and the addition of melamine resin (conservation). Thickness and volume increased significantly during this phase. After the drying process, the volumetric values (grey, yellow and blue line) decreased, especially the volume and thickness. However, slight dimensional changes in length and width were observed.

**Figure 5:**
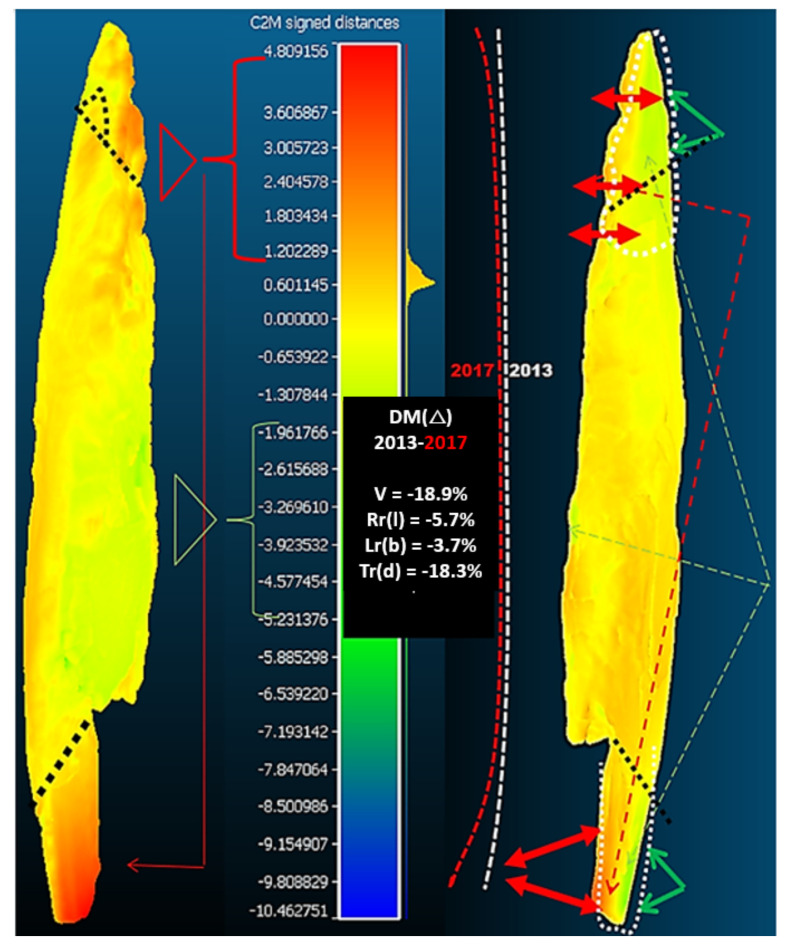
The deformation changes of the Palaeolithic wood point between the beginning and the end of preservation (volumetric changes, volume reduction and shape change) were calculated with the C2M (ICP) algorithm in the graphical software tool CloudCompare. The left image of the point shows the volumetric changes (red—bending of the upper and plant part; green—shrinking of the middle part) of the surface-based 3D model of 2017 compared to the reference 3D model (2013). The image on the right visualises a change in the shape of the 3D model of 2017 compared to the reference 3D model (2013). The colourimetric scale was created using an algorithm (C2M—CloudCompare) to statistically process the volumetric changes between the 2013 and 2017 models. The red-orange values represent the diffraction of the artefact from +3.6 mm to +1.2 mm. The blue-green values mark the shrinkage of the artefact between 10.4 mm and 1.9 mm. The data confirms the bending of the handle part and the top of the artefact. However, the shrinkage was more pronounced in the middle part. The cause of this deformation remains unclear. The unclear causes for the changes during the conservation process were the basic motivation for the creation of the anatomical 3D model of the Palaeolithic wooden point. By reconstructing the 3D model from 2D micro-CT images, we wanted to obtain volumetric data on the changes in the anatomical structure of the archaeological object.

Based on computer volumetric analysis of the five 3D surface models recorded in different years [4,7], it was found that there were significant changes and deviations after conservation (Table 1 and Table 2 and Figure 4). The artefact was visibly bent in the longitudinal direction (Figure 5). Strong bending was also observed in the plant part of the artefact. A slight bending of the artefact point was also confirmed by computer comparison. Its volume decreased considerably. The prevailing assessment was that the resulting surface changes were mainly due to the conservation process (swelling of the wood followed by rapid drying). A volumetric 3D model, which would reveal the internal structure of the wooden point, should answer the open questions about the changes detected in the analysis of the 3D surface models, such as bending, reduction in volume, change in shape, etc.

A comparative study of 3D surface models of the Palaeolithic wooden point has shown [5] that the 3D models recorded with optical scanners and reconstructed with conventional computer vision algorithms did not contain the necessary information to fully assess the condition of the artefact. The changes to the surface-based 3D models did not answer the question of what state the artefact was in after the conservation process, nor the question of the influence of internal deformations on the surface of the object. These shortcomings can be remedied with a volumetric 3D model that can be reconstructed from 2D tomographic images using computed tomography or computer vision algorithms.

A comparison of the amount of information contained in point clouds or triangulation meshes of 3D surface models acquired with optical scanners and tomographs illustrates the qualitative advantage of tomographs (Table 1). This also represents an important difference between the point clouds of the surface-based and volumetric models that can be reconstructed from tomographic images. Optical scanners provide only a small percentage of the information contained in the volumetric model compared to a tomography scanner. This fact is also confirmed by the finding that 3D surface models do not contain comprehensive information about an object. The difference between the two models is also that we can reconstruct both the surface-based and the volumetric 3D model from the point cloud obtained with a tomographic scanner. In contrast, only a surface-based 3D model can be reconstructed from the point cloud obtained with optical scanners (Table 2, Figure 4 and Figure 5).

Depending on the 3D scanner, the technique for reconstructing the 3D model is also different. In the traditional 3D surface model, we reconstruct the model by combining surface images (texture, volume, colour, etc.) in three-dimensional space. In the volumetric 3D model, we reconstruct a 3D model from the greyscale values (HU or RGB) of 2D tomography images. Such models are more accurate, of higher quality and with less noise compared to conventional computer vision models. They also provide optimal conditions for objective evaluation of the scanned object—volumetric analysis, qualitative analysis of the material, deformation analysis, etc.

### 1.1. Motivation

The Palaeolithic wooden point, which we have been studying since 2008, was scanned with a microtomographic scanner (Micro XCT 400) in 2018 and 2019. This provided 2D tomographic images that allowed us to look inside the artefact to better understand the reasons for the various deformations that occurred, especially after the conservation process. Analysing and understanding the changes in a waterlogged artefact over a period of 10 years is very valuable knowledge for scientists and conservators. Therefore, we decided to reconstruct surface-based and volumetric 3D models from the available 2D tomography slices. In this way, we were able to investigate the internal cracks, fractures, pores, inclusions and deformations of the wooden point. Since we wanted to have complete control over the tomographic reconstruction of the 3D model from CT sections, we decided to design and implement our own algorithm.

Reconstructing 3D models from CT images is a particular challenge for further research in computer vision, where rendering 3D models with open-source algorithms from images captured by DSLR cameras, structured light scanners, laser and other scanners is widely used for surface and remote sensing. This technique still belongs to the field of computer graphics. A number of segmentation algorithms are adapted to the specific objectives of the users. It is no coincidence that the primary application of noninvasive computed tomography is not the reconstruction of 3D models in the sense of computer vision. The focus is on the interest in noninvasive graphical insights, visualisations and analyses of 2D, 3D or 4D information in a three-dimensional coordinate system for the purpose of visual support in medical diagnostics, measurement technology, analysis and quality control of materials in industry and science.

Most algorithms for processing and reconstructing CT images have not received special attention from computer vision in general or its application in cultural studies due to the emphasis on commercialisation, closed code, specialisation (medicine or industry), radiation risk and intellectual property protection. With this novelty, i.e., the 3D anatomical model and the proposed algorithmic solution, we wanted to present a new challenge to traditional research and applied work in the field of computer vision in heritage sciences. The 3D anatomical model not only enriches the digitised cultural heritage and knowledge about the artefacts, but at the same time expands the possibilities for their analytical treatment, holistic visualisation and augmentation.

Since the background of our research team is in 3D computer vision [8] and we entered the heritage science by helping underwater archaeologists in documenting underwater sites [3,9], this foray into 3D models and tomography seemed promising. We aim to expand the research branch of computer vision with new experiences and insights—using CT, MRI and other depth imaging recording devices—from already-established surface 3D models to the reconstruction of anatomical 3D models and to the use of depth imaging recording devices, which are becoming increasingly affordable on the threshold of the fourth industrial revolution. We aim to complement traditional machine vision, machine and deep learning and artificial intelligence algorithms with noncommercial algorithms and software tools for reconstructing 3D models from CT images.

To reconstruct the 3D model from 2D images CT, we selectively used some time-limited open-source and commercial software tools (VolPack, ImageJ, Visualisation Toolkit, InVesalius, 3D Slicer, 3DimViewer and commercial software Avizo Fire, etc.) for 3D rendering and visualisation of tomographic images during the preparatory phase. However, we were not satisfied with the initial results. Our research objective was a comprehensive volumetric reconstruction of the internal (anatomical) structure of a 3D and 4D model of a selected archaeological object. Commercial 3D image processing programmes usually use special segmentation algorithms to process specific 2D image file formats and are designed for specific work and research goals in medicine or industry. However, they are not suitable for use in cultural studies. We often faced limited use of converted files, additional procedures, inaccurate volumetric data and substitution of 3D graphics for 2D graphics in analytical procedures. These circumstances were the reason why we decided to develop a special segmentation algorithm for reconstructing a 3D model from microtomographic images. The algorithm was adapted to the work requirements of archaeological and conservation treatment of smaller archaeological objects.

Archaeologists and conservators occasionally come across CT images of artefacts in their work, but they do not have the appropriate software tools and solutions for noninvasive reconstruction and analysis of 3D models. Since working with expensive hardware and commercial algorithms is not designed for the goals, interests, needs and problems of archaeological or conservation research, the vast amount of data and information contained in 3D anatomical models remains unused and undocumented. At the same time, the use of a customised segmentation algorithm also pointed to new challenges and opportunities in the reconstruction of 3D models in computer vision, where work is still based on more affordable hardware for reconstructions of only surface 3D models. Conventional 3D models of computer vision, compared to 3D models reconstructed from 2D images CT, are of lower quality, highly noisy, volumetrically unreliable and limited both graphically and in terms of data. We have successfully overcome these shortcomings with new, simple and undemanding segmentation algorithms for reconstructing a 3D model from 2D images CT.

### 1.2. Structure of the Article

The rest of the article is structured as follows: Section 2 gives an overview of the use of 3D modelling and computed tomography in archaeology, Section 3 deals with the state of the art in computed tomography in general, Section 4 deals with tomographic reconstruction in the context of considerations in archaeology, Section 5 describes the development of our own software for tomographic reconstruction. We have developed two versions of our software, the first with whole 2D slices CT and the second with segmented 2D slices CT. Section 6 presents the results of tomographic reconstruction with both versions of the software. The software was not only tested on the wooden point, but also on some other archaeological artefacts for which tomographic measurements (CT slices) were available. The internal structure of the wooden point was analysed in detail and various visualisations of this structure are given. The article ends with a discussion and conclusions.

## 2. Background

The London Charter [10] and the Seville Principles [11] recommended 3D models as the standard for archaeological documentation at the beginning of the 21st century. The introduction of new information technologies has fundamentally changed attitudes towards cultural heritage remains. The managers of archaeological collections are increasingly opting for 3D modelling, 3D visualisation or 3D augmentation of archaeological objects. In this way, archaeological objects are protected from the negative effects of ex situ conservation and public display. Archaeologists and conservators receive a more comprehensive professional treatment of the artefacts.

3D models reconstructed with traditional computer vision algorithms have become permanent digital carriers of artefact information that archaeologists can later examine, evaluate, compare and analyse without damaging or destroying the original objects. However, 3D surface models only contain information about the surface properties of artefacts. This information limitation can be improved by developing and using specialised algorithms to reconstruct volumetric 3D models of archaeological objects from image recordings of deep scanners (CT, MRI, PET etc.) to reveal their internal structure or anatomy as well.

The use of volumetric 3D models represents a qualitative advance in the integration of increasingly affordable computer technologies and software solutions into the regular professional and research work of archaeologists and conservators. The AGORA 3D Heritage Project has also been working towards this (The Belgian Federal Agora3D Project/BELSPO AG /00/164/—Royal Museum of Central Africa—Tervuren—Belgium) [12,13]

Surface-based and volumetric 3D models would greatly enrich archaeological documentation, improve work in archaeological laboratories and enable conservation science to deal comprehensively with cultural heritage remains. However, volumetric 3D models can provide conservators and restorers with additional useful information for planning the use of appropriate methods and techniques to protect critical cultural heritage remains.

### Computed Tomography for Archaeological Documentation

The number of scientific papers dealing with the use of computed tomography (CT) is very extensive in the international research environment. The number of bibliographic units has increased in recent years. This is due to the fact that investment in the development and use of CT technologies is increasing year by year and is becoming one of the most important areas of Industry 4.0 [14]. At the forefront is research into the use of CT in medicine, biology, chemistry, genetics and industry. Interest is also growing in the fields of civil engineering, materials analysis, cultural heritage protection and archaeology. A broader overview of the work of CT shows that the use of CT still raises a number of open questions that also directly affect its place and role in archaeology. These questions indirectly touch on the theoretical basis of our research, such as: optimisation of algorithms [8] for segmentation of CT images [15], development of specific protocols to achieve higher image contrast [16], revival of interest in the use of iterative algorithms in the reconstruction of 3D surface models [17,18], special control, steering and reverse engineering techniques in industry and additive manufacturing [19,20], inconsistent and poor standardisation of CT files [21,22,23], etc.

A review of published sources shows that the use of nondestructive CT or µCT technology in archaeology has so far focused mainly on the study of very delicate objects from the Palaeolithic and early civilisations. For example, CT scanners have been used to examine mummies [24], Ötzi [25] and The Venus from Willendorf [26], to read scrolls, to study the structure of clay tiles, pottery [27] and Etruscan bronze statues [28], to study textiles, wooden, bone and metal objects, the contents of sarcophagi, the forensic evaluation of art paintings, the technologies used to produce various objects and the mummification process, etc. Archaeologists still predominantly used the technologies, procedures and algorithms of medical computer tomography. It was only after 2015 that there was an awakening of interest in the development of special algorithms and in the use of specially adapted industrial CT readers for mapping earth layers at archaeological sites [29].

**Figure 6:**
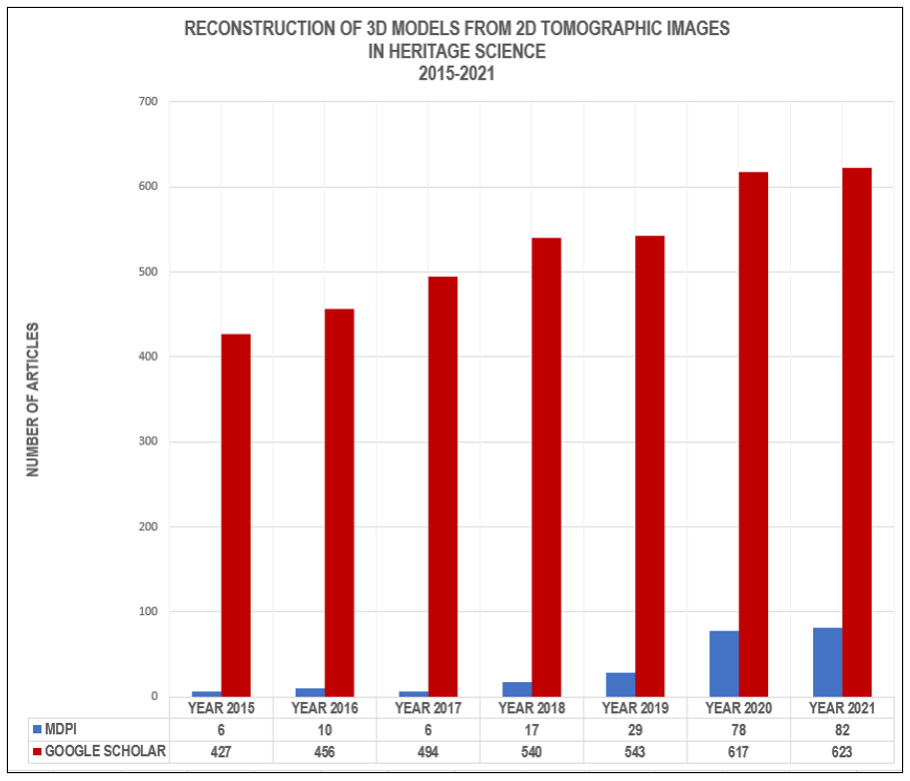
Number of articles indexed in Google Scholar and published in MDPI journals during 2015-2021/11 that represent the use of computed tomography in the field of cultural heritage science and are directly or indirectly related to the topic of 3D rendering from 2D tomography images or 3D slices. No article was dedicated to the problem of direct reconstruction of 3D models from 2D tomography images. The 3D models were reconstructed using commercial industrial tomography software (VGStudio MAX [31,34,35,37], Amira Avizo 9.0 [32,33], Simpleware—Synopsys, Inc, Dragonfly Pro—Carl Zeiss) and in most cases analysed from 2D slices in different layers. In no case did we record the use of any of the open source programmes to represent a 3D anatomical model. Open source programmes do not currently provide data for advanced statistical and geometric analyses.

After 2019, industrial µCT scanners and specialised commercial software will be increasingly used in archaeological studies (Figure 6). The first steps have already been taken to develop specialised µCT scanners suitable for [30,31] archaeological work. Archaeologists increasingly prefer the use of noninvasive computed tomography methods. With their help, archaeologists can analytically assess the internal structure and properties of smaller archaeological objects.

Current research interests in the noninvasive use, reconstruction, visualisation and processing of 2D µCT images include: analysis of all types of materials [32], analysis of the condition and production method of ancient Egyptian statues for restoration [31], morphological odontometric analysis of dental artefacts [33], visualisation of alloy composition and corrosion exposure of ancient Greek coins [34], evaluation of the suitability of µCT for the study of ceramic manufacturing technology [35], visualisation of the condition of clothing fabrics [36], analysis of manufacturing techniques and porosity levels of model samples of selected building materials (mortar) in historical objects [37], determination of the condition of the artefact before restoration [38], etc. An interesting example is the development of a system for the simultaneous use of computed tomography and photogrammetry [39] hardware for the visualisation of a 3D model. Important development and research work is being carried out in the computed tomography laboratory of the University of Bologna [30] and in the Centro Conservazione e Restauro “La Venaria Reale” to develop special tomographic devices for archaeological research. (CCR—Turin) [31].

The current importance and applicability of computed microtomography in analytical archeology is confirmed by the microtomographic treatment of the Venus from Willendorf by the Department of Evolutionary Anthropology and the Core Facility for Micro-Computed Tomography, University of Vienna, which researched and microtomographically processed the approx. 30,000 year old figurine made out of oolitic limestone using commercial computer software Amira. The research offered important new insights into the origins and methods of making this valuable archaeological artifact [26].

## 3. State of the Art in Computed Tomography

The original dilemma of algorithms for processing 2D images for CT at “low-level” was solved decades ago in the framework of Marr’s “high-level” paradigm [40], although computed tomography algorithms for processing X-ray signals and 2D images still have to deal with the problem of optimising edges and segmenting 2D images. The problem of robustness is obvious. Algorithms should primarily be based on the paradigm of active detection [41], i.e., they must be targeted at specific goals of detecting certain features, such as edge detectors, region growers, 3D recovery methods, etc.

The use of computed tomography and tomographic sensors is increasingly expanding the field of robotic surgery [42,43,44], artificial intelligence [45], computer graphics (e.g., 3D Geometry Generator Algorithm [46]) and computer vision (e.g., PT2PC model [47,48]).

**Figure 7:**
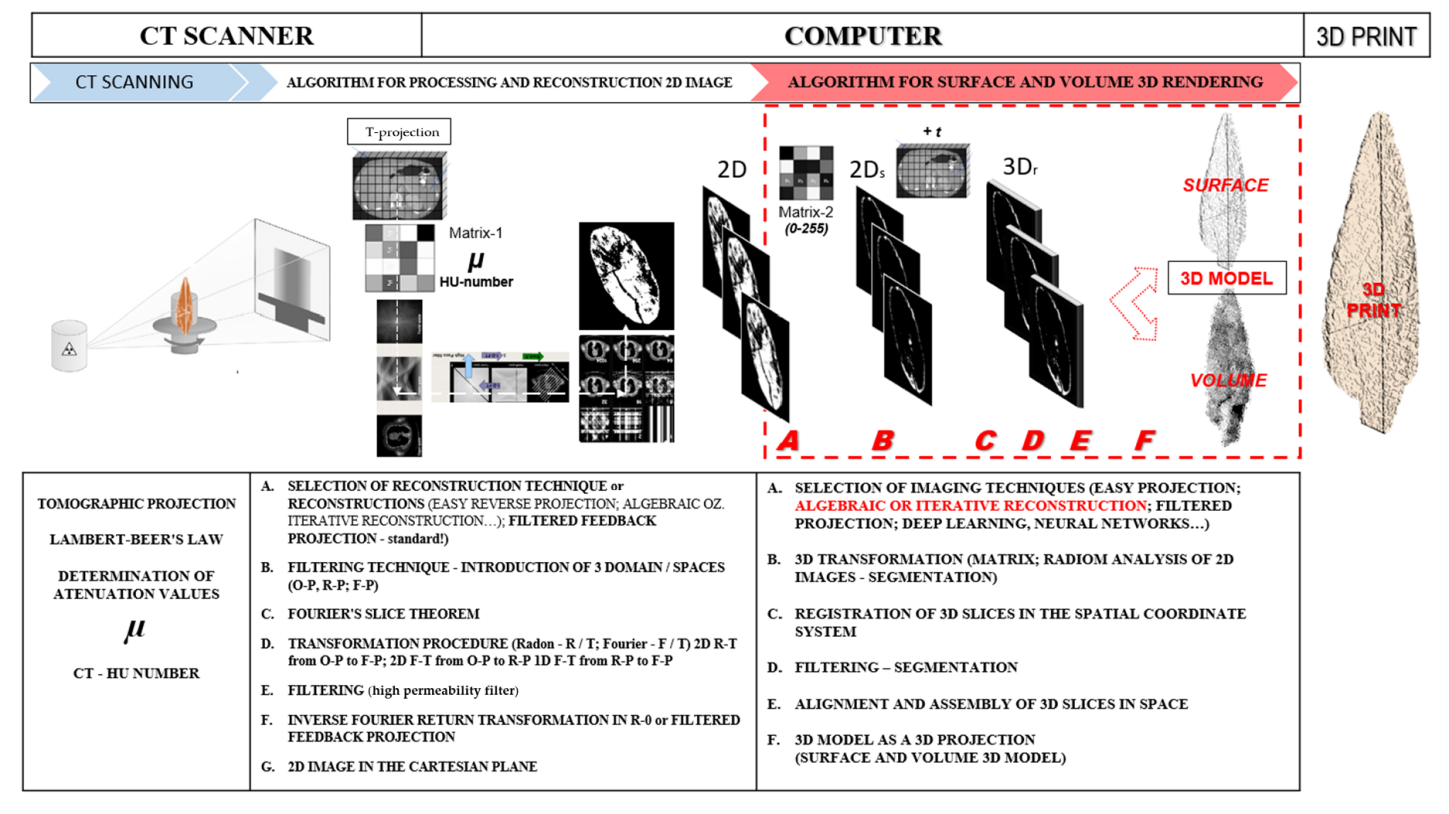
Computed tomography—presentation of the process of reconstruction of 2D CT images and 3D models.

In computed tomography we distinguish two types of algorithms, depending on the type of reconstruction. Figure 7 shows the process diagram of algorithms for spatial and surface imaging:

1. algorithms for the reconstruction of 2D tomographic images from tomographic projections and
2. algorithms for the reconstruction of volumetric 3D models from 2D tomographic images. For the reconstruction of 3D models from CT images, the terms “volume rendering” and “surface rendering” are often used synonymously in computer graphics [49,50].

However, the use of both terms requires some caution as they are often used uncritically and indiscriminately in professional articles, even in cases that do not involve reconstruction of 3D surface or volumetric models from CT, MRI or PET images and are based on attenuations or RGB matrices of grey values. The term “volumetric imaging” has not yet gained acceptance in computer vision methods and techniques for reconstructing 3D models. However, it is accepted in computer graphics methods and techniques for 3D visualisation of tomographic images [51].

While in the first case, the algorithms are adapted and specialised for processing image signals based on attenuation values or HU numbers, in the second case various specialised algorithms are used to represent the 3D surface model. These include algorithms from computer vision and computer graphics [52,53], such as the Marching Cubes algorithm, the dividing cubes algorithm, algorithms for visuomotor and haptic rendering [54].

The reconstruction of CT images in three-dimensional space is of particular importance in modern medicine and industry. Especially in robotic surgery, prosthetics and orthopaedics as well as in the analysis of material quality. This is not only about real-time visualisation and improving accuracy in the detection and treatment of diseases, such as in robotic surgery, but also about achieving higher quality products in industry. In both medicine and industry, methods for reconstructing and visualising 2D tomography images are on the rise. Algorithms for target rendering are also being adapted and developed accordingly. For example, Microsoft Visual Studio [49] has developed four algorithms with the Visualisation Toolkit (VTK) to help with robotic surgery: Marching Cube, Contour Filter, Composite Volume Rendering and Texture Mapping Hardware. Marching Cube and Contour Filter are algorithms for surface mapping, while Composite Volume Rendering and Texture Mapping Hardware are for spatial mapping. After 2000, similar development trends can be observed among other manufacturers of commercial hardware and software for computed tomography. The programming languages used to implement the algorithms are C++, Java and Python. However, it is undeniable that algorithms for surface or spatial imaging are adapted to specific needs and that the question of their robustness is still relevant.

In recent years, there has been increasing interest in the use of machine learning and deep learning algorithms, convolutional neural networks (CNN) or other artificial intelligence algorithms in industrial computed tomography. Occasionally, adapted or modified algorithms are used that have already been used in the reconstruction of 2D tomography images, such as Fourier volume rendering, Monte Carlo volume rendering [55], the additive re-projection technique, depth-shading algorithms, etc. [52].

Algorithms for imaging or reconstructing 3D volumetric models can be divided into four groups: Ray Casting Algorithms, Splatting Algorithms, Cell Projection Algorithms and Multi-Pass Resampling Algorithms. However, in the practice of 3D visualisation and modelling from tomographic images, three techniques for volumetric image projections in computed tomography stand out: maximum intensity projection (MIP), minimum intensity projection (MiniIP) and three-dimensional volume rendering (3DVR) [56] or direct volume rendering (DVR) [51].

An optimised 2D tomographic image is a key factor for the quality of all further procedures from visualisation to reconstruction of 3D models and addition. Therefore, most research efforts in computed tomography have been and still are focused on the improvement of algorithms for 2D image reconstruction and not on the reconstruction of 3D models. In computed tomography, computer graphics algorithms have become the most popular. Chris Shaw [57] mentions that so far (2014) only a modest 1.8% of research papers have been published that deal with CT and are also intended for 3D imaging.

A transparent contribution to the understanding and open questions of computerised tomography and the algorithms used for reconstruction was presented by G.T. Herman in his most cited work in the field of computerised tomography, his book *Fundamentals of computerised tomography: image reconstruction from projections* [58]. Herman describes how CT image data are obtained and used in science and medicine. The focus is on X-ray data, but also on the importance of CT in other fields, such as electron microscopy, nuclear medicine, ultrasound, magnetic resonance imaging, nondestructive testing and evaluation of materials, etc. A comparative evaluation of the reconstruction method and its accuracy under ideal and real conditions will be made. Reconstruction algorithms are also covered, including the filtered back projection, the extended Fourier theorem for reconstruction and back reconstruction, the linogram method for image reconstruction, algebraic reconstruction techniques, quadratic optimisation, etc. The book also draws attention to the open questions and problems of CT image reconstruction.

A second review of the features, capabilities and shortcomings of segmentation and reconstruction algorithms in computed tomography can be found in *3D Segmentation Algorithms for Computerised Tomographic Imaging: a Systematic Literature Review* [59]. The article includes peer-reviewed articles published in four scientific databases (Science Direct, IEEEXplore, ACMin PubMed) from 2006–2018. For the authors (Carvalho, Sobieranski and VonWangenheim), the key to the reconstruction process in computed tomography is the segmentation algorithm. A total of 182 papers were analysed, divided according to the methods and segmentation techniques used, namely thresholding methods, graph theory, level set methods, Markov Random Fields, active contours, flexible point distribution, wave densities, region growing, primitive shapes, use of filters and histograms, intelligent swarms, up to the use of convolutional neural networks, deep learning and machine learning. An interesting example is a method that combines segmentation and visualisation of multiple anatomical structures [60]. There is a very wide range of algorithms.

In tomographic image reconstruction in medicine, additive manufacturing, materials analysis and industrial control, the filtered backprojection (FBP) algorithm has been standardised for some time. In recent years, somewhat forgotten iterative reconstruction algorithms have resurfaced in industrial tomography [61,62,63,64,65]. Their use has become interesting with the increasing computing power of computers. Comparisons and research show certain advantages of the reconstruction algorithms over the FBP algorithm. New iterative reconstruction algorithms such as AIDR [62], ASIR and ASIR-V [66], IRIS [63,64] SAFIRE [65], ADMIRE [67], etc., show certain advantages over the FBP algorithm. Namely, iterative reconstruction significantly improves image quality due to cyclic processing. In medical tomography, iterative algorithms also mitigate the negative consequences (noise, artefacts, quality, sharpness) resulting from the requirement of selective use of the radiation dose index [62]. New iterative algorithms are already built into the latest generations of CT readers (e.g., Siemens, Toshiba, etc.) and are in most cases a trade secret.

The development so far shows a multitude of specialised reconstruction algorithms. The number and variety of algorithms is due to the fact that each selected algorithm and segmentation method or technique is adapted to specific research questions of 2D tomography image reconstruction in medical or industrial diagnostic imaging or analytical practise.

Computed tomography algorithm developers are still mainly focused on optimising algorithms for segmentation, recovery and 3D reconstruction of CT images [8,59] and on creating and using technological protocols or computational methods to achieve higher contrast and quality of CT layers in 3D visualisation [16]. However, research in iterative reconstruction (BIR, SiR-V, etc.) is still ongoing [17,18]. The transition from slice imaging to volumetric imaging and direct 3D reconstruction with spiral computed tomography (SCT) [68] has led to significant advances in medicine and industry in recent years. SCT also opens up new possibilities in the realisation of virtual archaeology projects.

## 4. Tomographic Reconstruction in Archaeology

Traditional radiology after 1889 and computed tomography after 1975 have been present in archaeology since its beginnings. The use of algorithms for the reconstruction of 2D images (for example: Inverse Fourier Transform or the filtered back projection commonly used today) is considered a noninvasive technique for the anatomical study of delicate, unstable and valuable artefacts [24] such as mummies, stone, Palaeolithic bone or wooden remains [4,5,7,69], papyrus scrolls, metal tools or weapons [28], jewellery, pottery, wall paintings, Ötzi—Italy [25,70], the Venus from Willendorf [26] etc.

Only rarely do we find examples of the reconstruction of archaeological 3D models from tomographic images. This is partly due to the development of a relatively autonomous field of computed tomography, which is primarily developed as an imaging technique in medical diagnostics and as a measurement and control technique in industry. The algorithms for reconstructing the X-ray signal into a 2D image and 3D visualisation from CT images are also geared towards these goals. Isolated attempts at 3D modelling from CT images are also the result of the high financial cost of using CT scanners and the still insufficiently exposed need to use 3D anatomical models in additive manufacturing.

The reconstruction of 3D models of archaeological artefacts has so far been mainly limited to 3D surface-based modelling in archaeology. 3D surface-based models have in most cases been created using affordable photogrammetry, laser scanners and structured light scanners. Various computer vision algorithms have been used for reconstructing 3D surface models, such as: intuitive algorithms for computing similarity or distance [71], SIFT—Scale Invariant Feature Transform [72,73], SURF—Speeded Up Robust Features) [74,75], ICP [76], SfM [76,77], SfS [78], SfL [79], segmentation algorithms [80], stereoreconstruction algorithms [81,82], self-learning algorithms [83], etc. In the last year, the use of artificial intelligence algorithms (deep learning, convolutional neural networks, etc.) has come to the fore. This is also the reason why the use of information technology in archaeology has focused on virtual archaeology [10,11], the additive production of copies of artefacts using surface-based 3D models and the digitisation of basic archaeological documentation. The processing, evaluation and comparison of the digitised information in archaeological document collections was left to future generations of archaeologists.

The reconstruction of anatomical 3D models from tomographic images is not yet the subject of interest of heritage sciences for a comprehensive documentation of archaeological objects. The use of computed tomography algorithms in archaeology still primarily adapts to the possibilities of arbitrary use of medical and industrial hardware. It is only since 2015 that we have seen greater interest from archaeologists and conservators in the use of computed tomography in the processes of conservation, restoration and visualisation of archaeological objects [84,85,86,87,88].

According to studies published in 2020 and 2021, archaeologists and conservators continue to use commercially tested material and composite analysis software tools to reconstruct or visualise 3D archaeological models, such as VGStudio MAX, Amira Avizo 9.0, Simpleware—Synopsys, Inc. and Dragonfly Pro—Carl Zeiss. We also recommend the possibility of online 3D visualisation of 2D images CT, with [89] interactive 2D and 3D graphics (WebGL), in any compatible web browser and without the use of plug-ins.

This is also the reason why, given the still financially inaccessible hardware, no specific computed tomography hardware or algorithms have been developed for the needs of heritage conservation, suitable for documentation and research science. Such initiatives have been presented in the past. For example, the Belgian project AGORA3D was launched in 2008. In recent years there have been efforts in this direction, for example by researchers at the University of Bologna and the Conservazione e Restauro “La Venaria Reale”—CCR of the University of Turin. An important innovation in the use of computed tomography for the conservation of valuable remains of cultural heritage are interdisciplinary projects of some French (e.g., the Introspect project) [84,88], British (RTISAD project), American (e.g., EDUCE project; Mummy project) [87], Canadian, Israeli, Austrian [86] and German [85] university research centres. In collaboration with specialised laboratories of state museums and some private companies, they use computed tomography (CT algorithms) in the planning, conservation and restoration of museum and archaeological exhibits.

## 5. Development of a New Tomographic Reconstruction Tool for Archaeology

The basic research objective was to select, improve and evaluate an algorithm for high-quality reconstruction of 3D surface and 3D volume models from tomographic images for the needs of preservation, conservation and evaluation of archaeological heritage remains. With the new approach in computer vision, mainly using algorithms for 3D surface-based modelling in the processing of 2D image records, we wanted to highlight the suitability and applicability as well as the analytical and documentary importance of 3D volumetric models in computer vision and heritage science. At the same time, we wanted to answer some open questions about the observed changes in 3D surface-based models of Palaeolithic wooden point from its discovery to the completion of conservation [2,4,5,7].

As open-source algorithms are used for observing 3D surface models obtained from image acquisitions from DSLR cameras, structured light scanners, lasers and other surface and remote sensing devices, reconstruction of 2D images from CT, MRI and other depth sensors also requires appropriate observational techniques. Most algorithms for processing and reconstructing CT images have not received special attention from computer vision in heritage science due to a focus on commercialisation, closed source, specialisation (medical or industrial), radiation risks and intellectual property protection. With new methods for reconstructing surface and volumetric 3D models from CT images, we aim to challenge the traditional research and application field of computer vision in heritage science.

With the new experiences, we aim to expand the research interest of computer vision (using CT, MRI and other depth-imaging scanners) from the already-established traditional 3D surface models to the reconstruction of surface models and 3D volumetric models from tomographic images. We anticipate that the price of 3D depth detection scanners will become more affordable for cultural scientists on the threshold of the fourth industrial revolution.

A particular research and development goal was to develop a simple algorithm for reconstructing volumetric 3D models from CT images of archaeological objects. The algorithm should not require highly specialised computer and mathematical knowledge from the user. We were guided by the idea that the algorithm should be simple, robust and adaptable to the specific needs of archaeologists and conservators. After all, they occasionally come across CT images of artefacts in their work, but they do not have the appropriate algorithms, customised software tools and solutions for reconstructing and analysing 3D models. Therefore, they are usually forced to use commercial software tools developed and adapted for other purposes, namely diagnostic work in medicine and industry. Since working with them is not tailored to the goals, interests, needs and problems of archaeological or conservation research, the huge amount of data and information contained in surface-based and volumetric 3D models remains unused. This leads to an impoverishment of the digital documentation of cultural objects. Developing a simple computer algorithm suitable for working with cultural heritage remains was our ultimate motivating goal, which would provide an incentive for further research in computer vision and cultural heritage science.

### 5.1. Design Considerations

After studying the literature and initially testing various computed tomography, computer graphics and computer vision algorithms for reconstructing 3D models from 2D images CT, we were faced with the need to develop a new iterative algorithm. The tested solutions did not meet our expectations to reconstruct a surface-based and volumetric 3D model that would answer the open questions about the identified changes in the five surface-based 3D models of the Palaeolithic wooden point accurately, volumetrically precise and graphically clear enough. The test with commercial software also showed that the built-in algorithms reconstruct the 3D model, but only the outer surface edge points of the cloud. Volumetrically, it reliably and accurately reconstructs only the 3D surface model of the scanned object.

The workflow of the new algorithm is based on a preliminary analysis of the properties of the 2D CT image slices and the projection of the output file of the 3D model (an OBJ file) based on the following parameters of the reconstruction of the surface-based and volumetric 3D model:

- 2D image CT in the format TIFF (JPEG, GIF, PNG, BMP, RAW, etc.) consisting of a grey HU or RGB matrix in which each point (pixel) of the matrix has a grey x/y value;
- the thickness of the X-ray beam corresponding to the *z*-coordinate value of each voxel of the 3D layer in Cartesian three-dimensional space;
- an edge-detection technique that segments selected greyscale RGB values of each boundary point (pixel) of a 2D image.

The algorithm must be adapted to the purpose of the archaeologist or conservator to reconstruct the 3D surface and 3D volume model of the artefact under investigation. In the selected test case this will be: detection of dislocations, inclusions, pores, cracks, openings, damage, deformations and fractures in the anatomical structure of the object.

The design of the algorithm should focus on the specific research objective of reconstructing the 3D surface and 3D volume model in the initial phase of development. Based on the available data contained in the greyscale values of the images from CT, we found that the reconstruction of the 3D spatial and 3D surface model can, on the one hand, use the full set of attenuation or RGB values and, on the other hand, develop an algorithm that can detect features in the anatomical structure of the object that are aligned with a specific archaeological or conservation goal.

Methodologically, two iterative algorithms were developed that differ in terms of the degree of robustness, the research purpose and the temporal dynamics of the implementation:

- the algorithm with the working code name *dAR3D* converts 2D images from CT directly and without additional segmentation into 3D slices CT (3D scalar field of voxels), registers them in a three-dimensional coordinate system as a collection file and then reconstructs them into a volumetric and surface-based 3D model, and
- the algorithm with the working code name *sAR3D*, which first limits the number and values of features in the 2D image CT by segmentation. It then converts the segmented 2D images CT with a *z*-coordinate value into a 3D scalar field of voxels (3D CT slices), registers them in a three-dimensional volume coordinate system as a collection file, and then reconstructs them into a 3D volume and 3D surface model using the aggregation and alignment method. The segmentation algorithm is intended for specific analysis or research goals in the archaeological, conservation or restoration treatment of an archaeological object. It is faster than the *dAR3D* and can be implemented on personal computers without memory limitations. It can also be used in other fields or in cases where we decide to segment features in a 2D CT (MRI etc.) image.

The algorithms are limited to the process of reconstructing the 3D surface model and the 3D volume model from 2D images CT (Figure 7).

The direct algorithm *dR3D* is slower (due to the large amount of information contained and consequently the size of the output file). Reconstructing a 3D model takes about 7 times longer than reconstructing a 3D model from segmented CT images. Due to the large amount of information, additional computer storage capacity or cloud processing is required. This algorithm is useful:

- in the initial nonselective and noninvasive examination of the anatomical structure of the artefact to obtain initial information about the artefact and for the subsequent selection of target features for archaeological, analytical or conservation processing;
- for a comprehensive archaeological 3D documentation, and;
- to produce a 3D additive as a perfect replica of the original.

The segmentation algorithm *sAR3D*, which was the subject of our special development refinement due to its robustness, simplicity and efficiency, is adapted to the research goals of the direct user. It is intended for the working purposes of archaeological, conservation or restoration treatment. It is faster in time and can be realised on noncommercial personal computers without memory limitations. It is user-friendly, robust and simple. No special knowledge (radiology, mathematics, physics, computer vision, computer graphics, computer tomography, etc.) is required to achieve the user’s research goals of reconstructing a 3D model from CT images.

**Figure 8:**
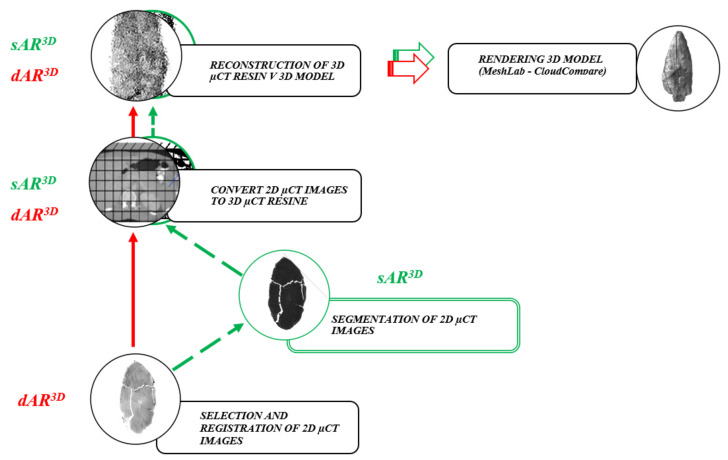
Workflow of the direct *dR3D* and the segmentation algorithm *sAR3D* for reconstruction of 3D models from CT images.

The workflow of the two algorithms is shown in Figure 8.

### 5.2. Implementation

Code the algorithms for direct and segmented reconstruction of 3D models from 2D µCT (or any 2D images obtained with CT, µCT, nano- CT, MRI, MMG, ultrasound or other depth sensor readers) in four steps. The individual steps and specifics of the code record structure are shown in the tabular overview (Table 3), which lists the basic code records.

In the mathematical and logical design of the code of both algorithms, we used a numerical analysis software package, MATLAB. The chosen fourth-generation programming language is sufficiently effective for performing mathematical operations. However, the segmentation process is only one form of mathematical algorithm. In addition, the programming language can quickly work with tables to quickly convert segmentation data into a 3D coordinate table.

The code of both algorithms is the same in the first step (selecting and registering 2D images CT for 3D reconstruction), in the phase of converting 2D images into 3D layers and in the phase of writing a 3D model in OBJ format. The codes of the two algorithms differ only in the fourth step, where we use the outer and inner edge detection technique to segment individual 3D layers.

The code uses the auxiliary algorithm of the NATSORTFILES function (built into MATLAB SORT), which classifies an array of cells with filenames or paths. It takes into account all numeric values in the array (instead of the default sort, it uses “natural sorts”). It is a “natural sort type” or an “alphanumeric type”. NATSORTFILES (74) does not mean sorting by natural order, but separates file names and their extensions. This has the effect that shorter file and directory names are always placed before longer names, thus ensuring the correct order of names in the directory. For the same reason, file paths are divided into each path delimiter and each directory level is sorted separately.

A special feature of the code of both algorithms is the input of “z-values” or “z-coordinates”. This procedure is crucial for the correct volumetric arrangement of the 3D model. If we want to reconstruct a surface and volumetric 3D model from CT images that allows accurate volumetric analysis and comparison, it is not enough to simply fold 2D images into a virtual 3D model. To achieve a volumetric match with the original, each 3D layer must be inserted into a Cartesian coordinate model of the space with precise voxel dimension data (the “z-coordinate value” in the 3D model space). Only in this way can we obtain a volumetrically accurate 3D anatomical model that is volumetrically identical to the original. If we simply glue the images of CT together, we will obtain the correct values for the width (x) and thickness (y) of the object, but the length (z-coordinate) will be completely distorted, unrealistic and useless for volumetric analysis and final reconstruction.

The fundamental difference between the two algorithms lies in the introduction of the segmentation procedure, which in our case is based on the technique of detecting edges on the surface and in the anatomical structure of the studied object. The segmentation procedure removes insignificant grey values of edges from the image CT and determines the edge values that will be the subject of the user’s examination. The segmentation procedure allows us to highlight the selected target features of the anatomical structure of the original more clearly in the reconstruction of the 3D model than with the dAR3D algorithm. In this way, we simplify and shorten the reconstruction and extract the features highlighted in the 3D anatomical model while preserving all volumetric values and information.

### 5.3. Segmentation

In our case, segmentation is considered as a form of mathematical algorithm. This was also the reason why we chose a numerical analysis software package (MatLab) to write the code. This programming language is suitable for working with tables and can effectively convert segmented data into a table with 3D coordinates. The code written in this programming language is generally robust, easy to understand and use, optimal for processing image files (e.g., TIF, JPEG, PNG, etc.) and does not require additional and intermediate procedures.

**Figure 9:**
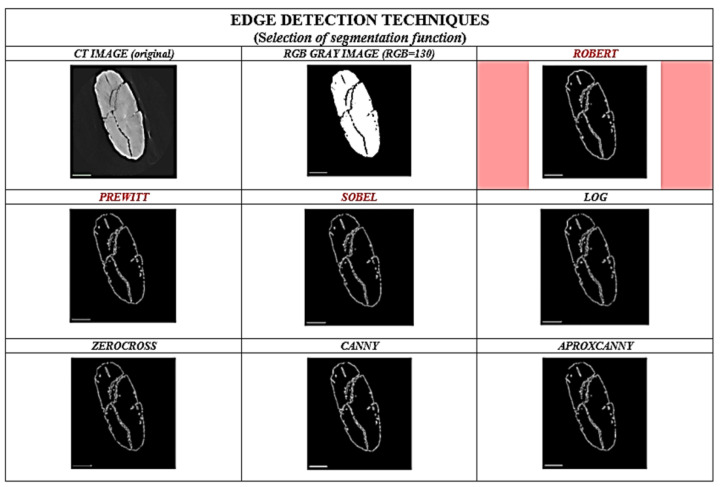
Comparison of different edge detectors for segmentation of 2D slices CT. The figure shows the test results of the different edge-detection methods. The analysis and comparison of the test results was the basis for selecting the most suitable operator for performing the segmentation process in the phase of preparing the 2D micro-CT images for placement in 3D space and reconstruction of the 3D model. The Roberts Edge Operator was selected as the most suitable operator for performing the segmentation process. The advantage of the Roberts segmentation function is that it maintains the most small details without altering small cracks or indentations (these differences are more noticeable at higher magnifications).

The right choice of edge-detection techniques is crucial for efficient segmentation. The edge of a CT image can be defined as an abrupt change in intensity. The edge is nothing more than the boundary of a particular region. In a CT image, the edge can be defined as a break in brightness or a change in the grey values of the HU or RGB matrix. When the light intensity or the grey value changes, this is called an edge. Edge perception is important for describing the shape and anatomical structure of an object. Edge detection identifies boundaries in a 2D image CT. The features can be common or separate. The main goal of edge detection in the segmentation algorithm is to obtain the volume and surface features of the object. Before selecting the segmentation code, we investigated and evaluated different edge detection techniques (Figure 9) and selected the most suitable one for reconstructing a 3D model of an archaeological object.

We have investigated, compared and tested different techniques (Roberts, Prewitt, Sobel, Log, Zerocross, Canny and Approximate Canny) for edge detection in the image CT of the Palaeolithic wooden point and some other artefacts (Figure 9). We chose Roberts edge-detection technique, which we found most suitable for processing CT images of archaeological artefacts. This technique is simple, robust and does not smooth edges compared to other edge detectors. It is also capable of detecting smaller openings. It uses a 2 × 2 convolution mask. The Roberts operator [90,91,92] is widely used for image processing in computer vision. It is one of the oldest operators and was introduced by Lawrence Roberts in 1963. The operator is also known as Roberts’ cross operator. The edge image can be calculated quickly and easily. Although it is the oldest and simplest, it has proven to be the most reliable in reconstructing a 3D model of an archaeological object compared to other techniques.

The process of reconstructing the 3D surface model and the 3D volume model from CT sections was adapted to the specific research objectives of the *archaeological or conservation treatment* of the artefact using the segmentation *sAR3D*. Segmentation in our case is a technique for determining, selecting and limiting the grey RGB values in CT images. This also allows us to reconstruct the 3D surface and 3D volume model. It is a rarely used 3D modelling technique, even when it comes to the commercial reconstruction of a 3D model from CT images. However, CT image segmentation is different from the prevailing 3D modelling techniques in computer vision, where segmentation is based on other paradigms, such as: light sources, contrast, colour shading, generalisation of geometric shapes, etc.

In our particular case, segmentation was used to determine and mark the edges of the non-oody x–y–z coordinate points (voxels) in each 3D layer. This marked the features in the anatomical structure of the 3D layer (the outer edge of the artefact, the edges of the anatomical—in our case—nonwoody features of the object). We selected those features in the 3D layer which, in accordance with the aim of the study, we believed could successfully answer the question about the nonwoody features of the artefact and the factors that might influence the observed surface deformation of the object. In our case, these were openings, pores, fractures, inclusions, cracks, etc. By determining the outer edge handles, we have set the stage for the reconstruction of the 3D surface model, while the 3D anatomical model will volumetrically mark and represent selected features that were the subject of research interest and observation.

### 5.4. Visualisation of Results

The visualisation of the 3D model, which was reconstructed from 2D images CT, is done with the graphical open-source software tools CloudCompare and MeshLab, which enable high-quality and accurate visualisation of 3D models, their volumetric analysis and comparison.

**Figure 10:**
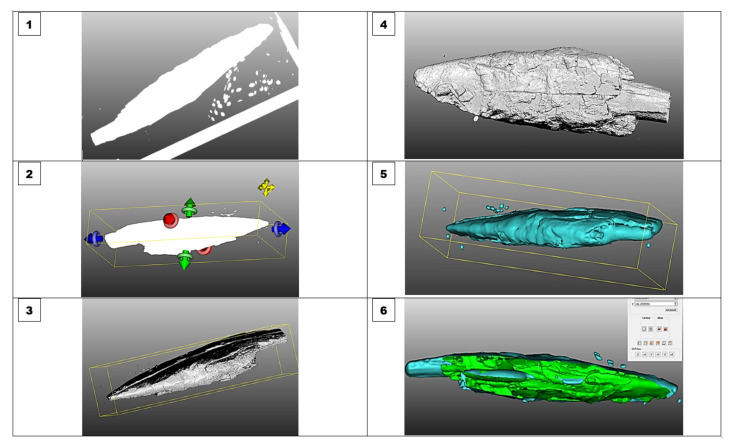
3D model visualization process in the CloudCompare software tool. Read the explanations of the six steps in the text of the article.

The visualisation process is simple. It is done in six steps using the tools, methods, techniques and algorithms built into the software. The following is a summary of the main procedures for visualising 3D models with CloudCompare (Figure 10):

**Step 1:**  the point cloud of the 3D model in the selected format (OBJ, PLY, STL, etc.) is entered using the “Open” function in the CC software;
**Step 2:**  The tool CROSS SECTION removes unwanted or disturbing sections in the point cloud and then exports the selected section as a new point cloud to CC;
**Step 3:**  The calculation of NORMAL with the surface approximation method follows (it is possible to select the methods depending on the goal of the visualisation: planar; triangulation; square);
**Step 4:**  The calculated normals are aligned to the normals using an orientation algorithm or a numerical Fast Marching method (or using the Minimum Spanning Tree algorithm);
**Step 5:**  Poisson Surface Reconstruction is used to convert a point cloud into a 3D model with triangulation mesh.
  Depending on the exposed research objectives and the planned treatment of the 3D model and the objectives of its visualisation, follows.
**Step 6:**  For further processing and clearer visualisation, various filters, stereogram analyses and other tools are available for multilevel volumetric, statistical and analytical treatment of the point cloud (e.g., cross sections, 3D depth views, comparisons, smoothing, volumetric and statistical tools, segmentation, scalar fields, etc.). In our case, we opted for anatomical or volumetric 3D visualisation and volumetric detection (*x*, *y*, *z*) of critical points in the anatomical structure of the Palaeolithic point.

By processing the triangulation mesh of the points of the visualised model, we can successfully perform an accurate volumetric and deformation analysis and comparison (deformation monitoring) of the selected 3D models.

## 6. Results

Algorithms for reconstructing 3D models from CT images were tested on a 40,000-year-old Palaeolithic wooden hunting weapon found in the Ljubljanica River (Slovenia) in 2008 [2]. To obtain an assessment of the quality of the represented 3D volume and surface model, the algorithms were tested on four other archaeological objects (bone flute from Divje babe I [69] and three ceramic rattles from the Bronze Age [93]). The robustness of the algorithms was also tested on two composite materials (concrete and fabric). The quality of the reconstructed 3D models was compared with models visualised with the commercial software tool Avizo Fire (FEI).

Volume and deformation analyses with the software CloudCompare were carried out for 3D volumetric models of the Palaeolithic wooden point.

### 6.1. Input-Output Data for the Reconstruction of 3D Models from CT Images

Algorithms used for reconstruction of 3D models from CT Images:

- *dAR3D*—direct algorithm for 3D model reconstruction;
- *sAR3D*—segmentation algorithm for 3D model reconstruction.

Input data (Table 4): reconstructed two-dimensional microtomographic images of an archaeological object in the format TIFF, created on the basis of a matrix of attenuation values (HU -number) and filtered back-projections with a MicroXCT 400 scanner.

Output data (Table 5): 3D model in format OBJ. Displayed and processed with the software tools MeshLab and CloudCompare.

### 6.2. Hardware and Software

The algorithms were systematically developed, tested and evaluated on a commercially available portable computer (a laptop) with the following configuration:

- PROCESSOR: INTEL (R) Core (TM) i7-8850 U @ 1.80 GHz;
- RAM: 8 GB;
- GPU: NVIDIA GeForce RTX 1050;
- OS: Windows 10 (64-bit).

MatLab (ver. R2018a—a custom multiparadigm programming language and numerical computing environment) was used to develop the algorithms.

Open source applications for graphical, statistical and volumetric processing MeshLab and CloudCompare were used to process image data, compare and reconstruct the 3D model.

### 6.3. Reconstruction, Comparison and Analysis of 3D Models of the Palaeolithic Wooden Point

**Figure 11:**
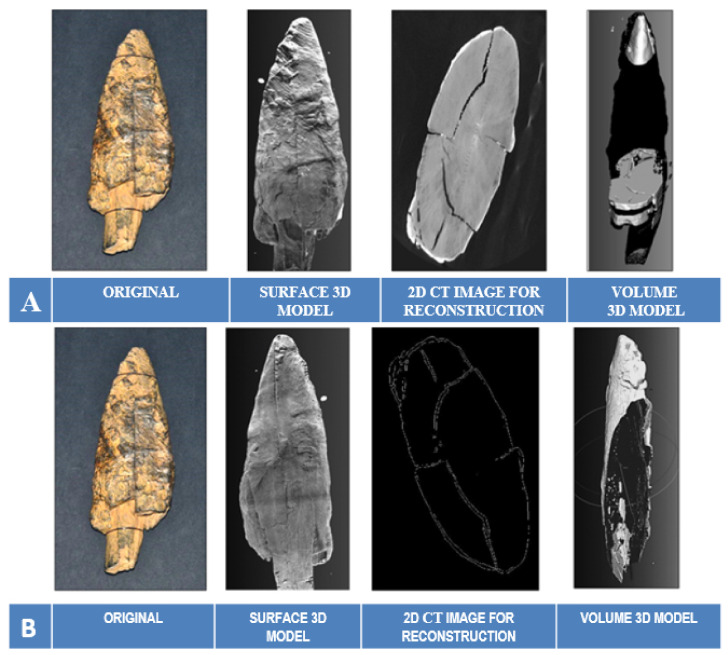
Reconstruction results: (**A**) direct algorithm *dAR3D*, (**B**) segmentation algorithm *sAR3D*.

The reconstruction of the 3D models of the Palaeolithic wooden point was carried out using both developed algorithms (Figure 11). Based on the obtained results and volumetric data of the point cloud in 3D Cartesian space, we performed an analysis of the anatomical structure and properties of the 2019 3D volumetric model and compared the obtained data and information (deformation monitoring) with the 2018 model. The visualisation and comparison were performed with the graphical software tool CloudCompare.

#### 6.3.1. Reconstruction with Algorithm *dAR3D*

**Figure 12:**
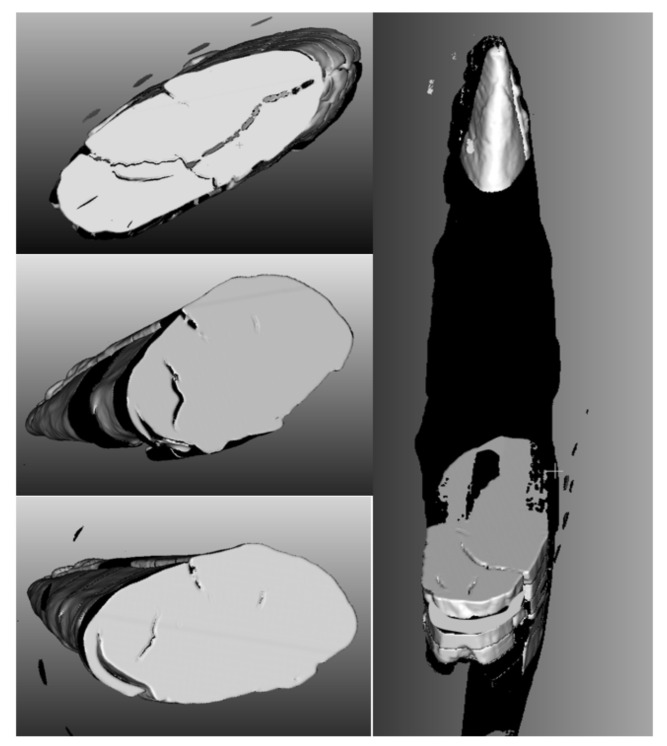
Example of a 3D model reconstruction with the algorithm *dAR3D*. The model detects anatomical changes (cracks, fractures, etc.), but the anatomical structure is also filled with woody parts. The reconstruction of the 3D model was performed considering all RGB values (0–255) of the grey matrix of the 2D images. Deformations of the internal structure of the model are only visible after individual sections. The model does not provide a detailed 2.5D insight into the artefact despite the large amount of information and data.

Using the direct algorithm (*dAR3D*), we reconstructed the 3D surface-based model and the 3D volumetric model directly from the entire set of 2D images CT of the Palaeolithic wooden point (Figure 12) as captured in the HU matrix by the CT reader and recognised by the RGB matrix.

Compared to the original and the model reconstructed with the commercial tool Avizo Fire (FEI), the surface-based 3D model is extremely accurate and even surpasses the photo of the original in certain parameters (contrast, properties of the surface texture). The difference in contrast and accuracy between the reconstructed 3D model and the original photo is due to the limitations of the DSLR camera such as colour gradations, the influence and direction of light on the artefact and other circumstances of the photo. The difference in contrast between the reconstructed dAR3D model and the model displayed with the commercial Avizo Fire (FEI) tool is due to the use of different 2D image resolutions in the TIFF format. In our case, the image resolution used to reconstruct the 3D model was significantly higher (2690 × 2731) than the image resolution (1012 × 1024) used by the commercial tool to display the 3D model. This is also the reason why our model is more accurate and the point cloud also contains a much larger set of stored information.

#### 6.3.2. Reconstruction with the Segmentation Algorithm *sAR3D*

**Figure 13:**
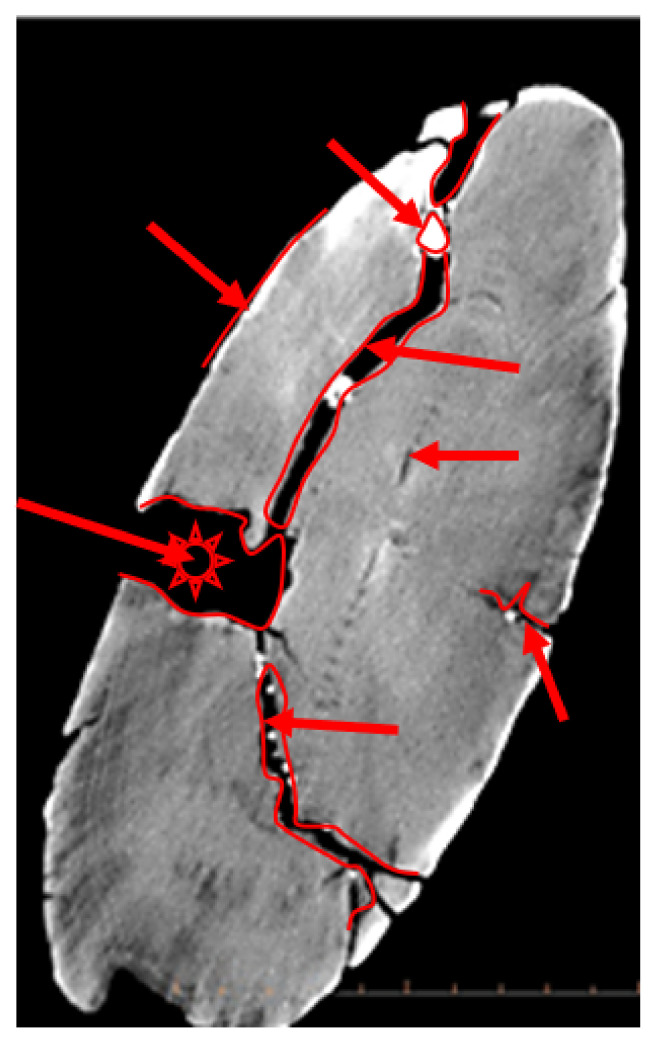
In the 2D CT image the edges of larger openings, fractures, pores, inclusions, etc., are detected first.

For the implementation of the segmentation algorithm *sAR3D*, we first marked only the grey RGB values (−278 HU; 110–130 RGB) in the 2D images CT for the reconstruction of the 3D surface model and the 3D volume model, as well as regions that indicated nonwood features or values in the images. With this intervention, we limited the segmentation to selected features in the anatomical structure of the point (Figure 13), such as dislocations, inclusions, pores, cracks, openings, damage, deformation and fractures. According to archaeologists, conservators and restorers, these deformations are important for planning procedures to protect artefacts and for further 4D analyses (deformation monitoring) of 3D volume models of the point (2018 and 2019).

**Figure 14:**
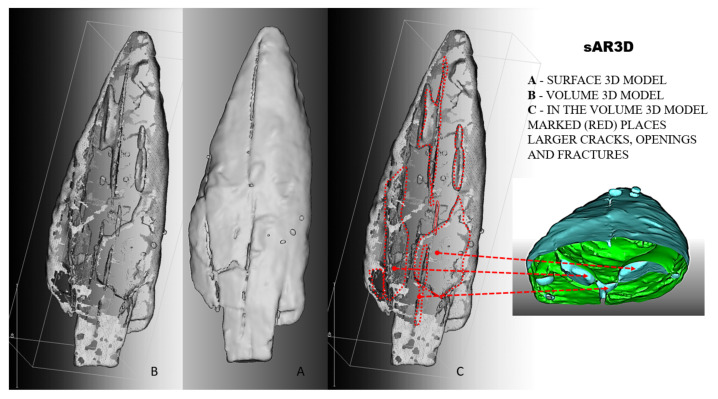
Palaeolithic wooden point: (**A**) 3D anatomical (volume) model, (**B**) 3D surface model, (**C**) 3D anatomical model with marked deformations (red dashed lines indicate the outer edges of cracks, openings and fractures in the internal structure of the point). The segmentation algorithm provides a 2.5D insight into the anatomical structure of the artefact after reconstruction. The outer surface boundaries of the artefact are marked in blue, with the light blue representing the inner openings, cracks and other deviations. The green colour represents the inner boundaries of the woody part of the artefact.

The properties of the surface model reconstructed with the algorithm *sAR3D* are identical to the results described and summarised in the model presented with *dAR3D*. Since the segmentation algorithm limited the set of point clouds and the target information in CT images of the point’s anatomical structure to the RGB values of the nonlignified parts of the point (pores, openings, cracks, fractures, etc.), only the segmented values in the anatomical structure were highlighted in the 3D volumetric model (Figure 14). The model accurately and clearly represents the changes, features, characteristics and deformations of the artefact.

### 6.4. Comparison of the Quality of Surface-Based 3D Models of the Palaeolithic Wooden Point

To determine the efficiency of the algorithm and the quality of the reconstructed 3D surface-based model, we compared the 3D model of the wooden point reconstructed with the algorithms *dAR3D* and *sAR3D* with the 3D surface-based model of the point reconstructed with the commercial software package Avizo Fire (FEI). The comparison (graphical and statistical) was performed using the ICP algorithm and the CloudCompare graphical software tool. The comparison showed slight discrepancies between the two models. From this comparison it can be concluded that the quality of the reconstructed 3D model depends mainly on the quality of the input data. This fact also became clear when comparing two models that were created at different resolutions. Here the differences were in the contrast and texture details of the surface-based 3D model and not in its volumetric deviations.

### 6.5. Anatomical Characteristics of Surface-Based and Volumetric 3D Models of the Palaeolithic Wooden Point (2018–2019)

In the volumetric computer analysis of the anatomical structure of the volumetric 3D models, we restricted ourselves to a model reconstructed with a segmentation algorithm *sAR3D*. During segmentation, we paid attention to dislocations, inclusions, pores, cracks, openings, damages, crisps, deformations and fractures. We located critical areas, identified damage and changes that were previously not readable from the 3D surface-based model.

**Figure 15:**
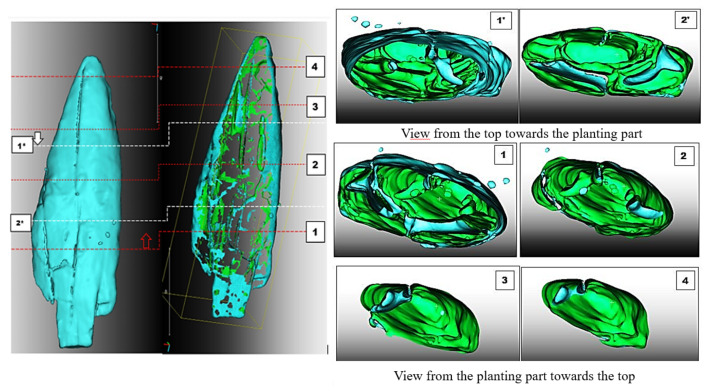
Exposed critical points in a 3D volumetric model of a Palaeolithic wooden point. A blue-green grid was chosen to make the anatomical structure of the artefact clearer. The light blue colour indicates the outer surface boundaries of the artefact and the inner boundaries of the non-wooden deformations (openings, fractures, cracks, pores, etc.) in the anatomical structure. The green colour indicates the inner boundaries of the wooden part of the artefact. The images show a view of the inner structure from the tip to the handle part (1’ and 2’) and from the handle part to the upper part of the artefact (1–4). Deviations and critical points are clearly visible.

**Figure 16:**
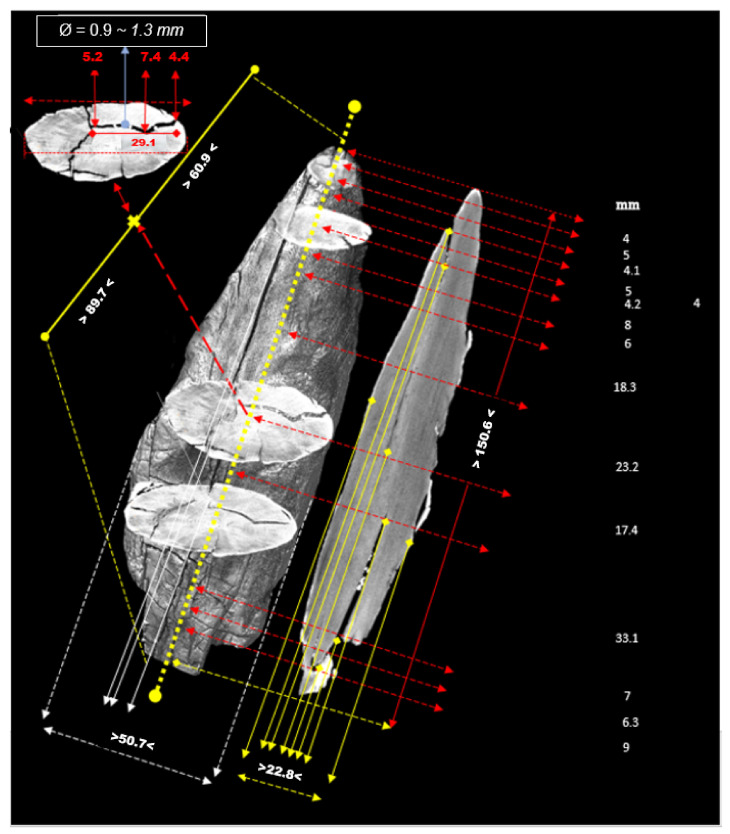
Volumetric microlocations of critical sites in the volumetric 3D model of the Palaeolithic wooden point.

**Figure 17:**
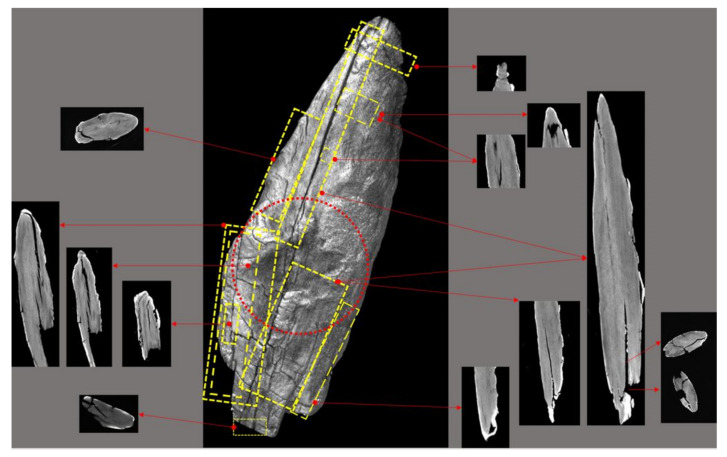
Overview of the critical points in the volumetric 3D model of the Palaeolithic wooden point.

**Figure 18:**
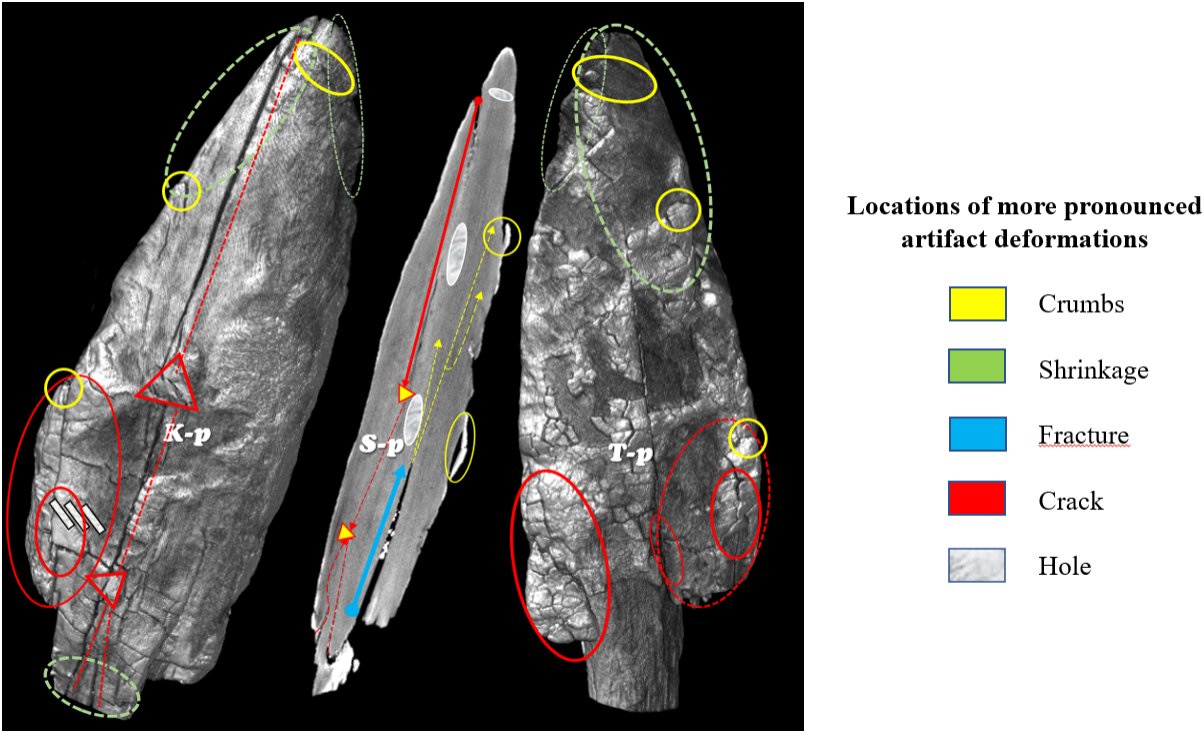
Locations of exposed deformations of the Palaeolithic wooden point in the volumetric 3D model, which was recorded with a µCT scanner in 2019.

**Figure 19:**
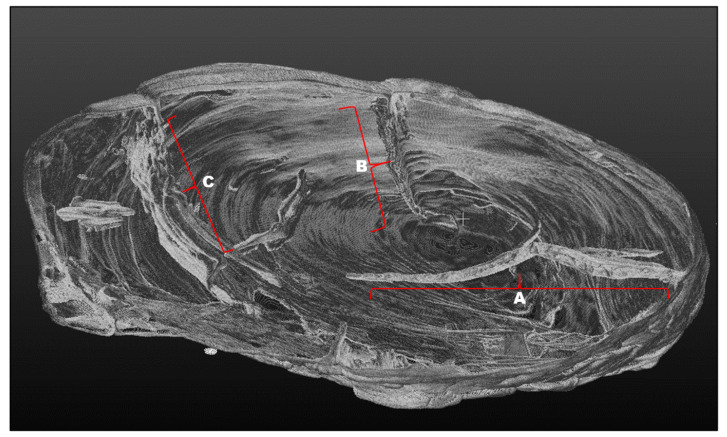
A three-dimensional depth image (viewed from the handle section) of the exposed critical areas in the anatomical structure of a Palaeolithic wooden point. Three main deformations were noted in the anatomical structure: a crack (B) running the entire length of the surface of the artefact; a transverse fracture (A) extending from the sampling point to the centre of the artefact; and numerous deformations, fractures, pores and cracks in the left wing of the artefact (C).

**Figure 20:**
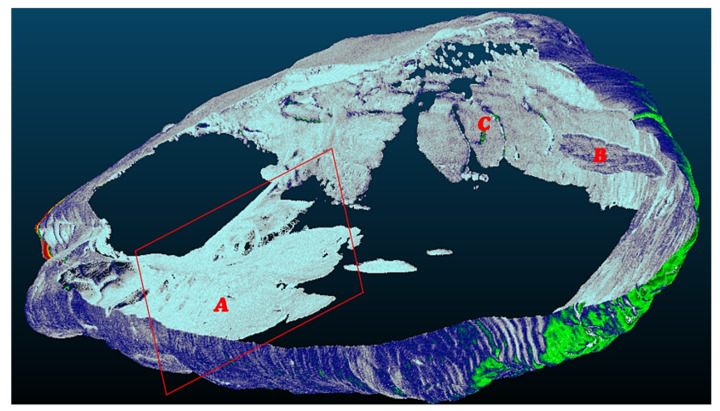
Fracture (A), which runs from the junction of the socket part and the point into the interior approx. 4.7 cm. A longer opening (B) is visible inside and cracks and fractures (C) in the left wing of the Palaeolithic wooden point.

The critical points of the artefact’s internal structure were determined volumetrically and marked on the model (Figure 15, Figure 16, Figure 17 and Figure 18). Three pronounced internal deformations were detected (Figure 19 and Figure 20): an elongated crack (B), a pronounced fracture (A) and a deformation (C). These three internal deformations stimulated the bending of the upper and lower part of the point after the conservation process, which was detected in the volumetric analysis and the comparison of the 3D surface models.

**Figure 21:**
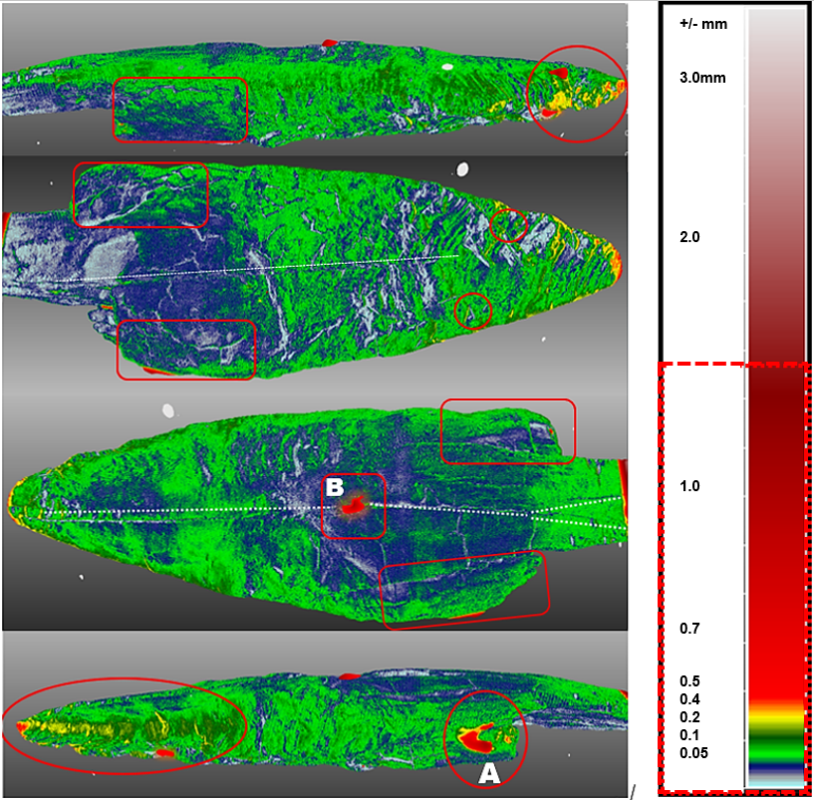
Changes in surface deformation of the 2019 3D model of the Palaeolithic wooden point, compared to the 2018 reference model (changes are in a limited range between 0.0001 and 1.5001 mm). The colour matrix scale of deformation monitoring of the 2018 and 2019 3D models confirms the one-year dynamics of surface changes. Compared to other 3D models (2009–2017), the dynamics of changes on the artefact surface has stabilised. The shrinkage of the artefact persists with an average of 0.1 mm per year (green grid). However, the deformation of the uppermost point is even more pronounced. It lies between 0.5 mm and 1.0 mm (red-orange-yellow grid). The annual bending of the top by 1.35 mm is confirmed volumetrically. The bending is detected in the area of the handle. This has bent by 1.7 mm compared to 2018. More deformation of the left wing (A) of the point was also noted. In this area, the anatomical model drew attention to a number of unnatural internal cracks and deformations. Deformation variations in this area ranged from 1.1 mm to 2.2 mm compared to 2018, and the crack in the central part (B) widened by 1.4 mm. At the exposed point (B), it reaches a depth of 7.1 mm. Monitoring of the deformation was carried out using the CloudCompare software tool and the C2M algorithm (ICP).

**Figure 22:**
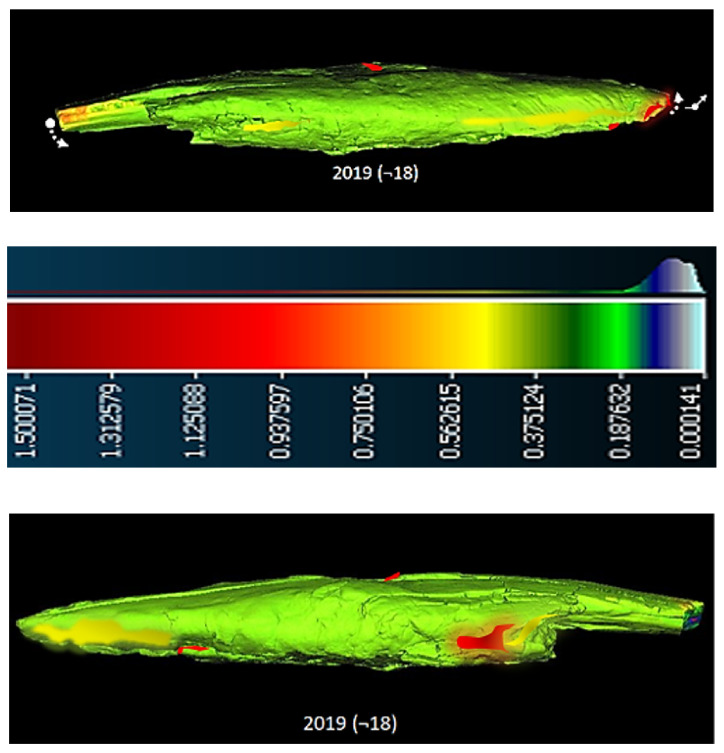
Exposed sites of changes in the surface-based 3D model of the Palaeolithic wooden point (2019 —comparison with the 3D model from 2018). Volumetric measurements confirm the calming of the point deformation process. It is still dominated by shrinkage or bending in the range of 0.18—0.37 mm. Stand out (red value on the deformation scale—from 1.2 to 1.5 mm) deformation changes in the top, planting part and left wing point.

A comparison was made between models of the Palaeolithic point reconstructed in 2018 and 2019 using a CT scanner (Figure 21 and Figure 22). Compared to the 2018 reconstructed model, the length of the point has shortened by 1.3 mm (−0.84%) in 1 year due to changes in deformation (shrinkage and bending of the point). The width and thickness at the most exposed points have increased—due to the expansion and deepening of the internal cracks—by 2.4 mm or 1.6% (width) and 1.1 mm or 0.73% (thickness), respectively. The internal dynamics of the surface changes show a tendency to settle down in this relatively short time interval. This fact underlines that the interval of the conservation process is longer in time than its official conclusion. The artefact is a “living organism” responding to altered ex situ nutritional conditions.

**Figure 23:**
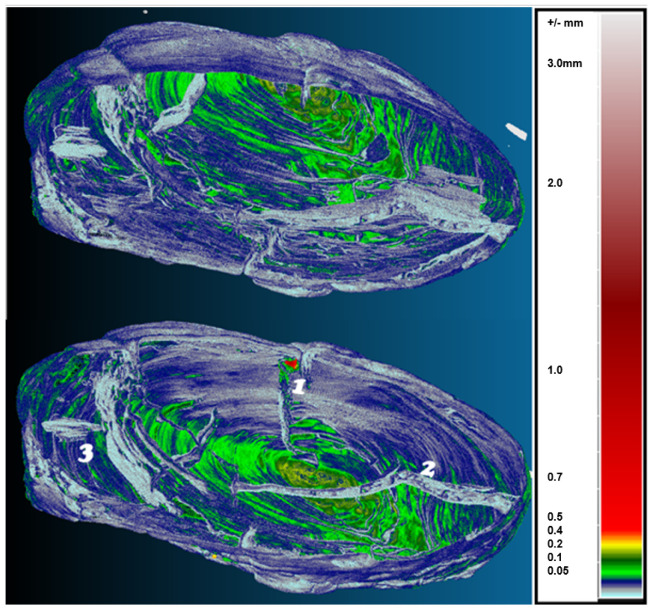
Annual dynamics of changes in anatomical structure (model—2019; reference model—2018)—view from the grip area to the top of the Palaeolithic wooden point. The deformation changes in the anatomical structure are clearly visible (crack along the entire length of the upper part of the artefact (1); larger fracture (2) in the lower and middle part; numerous unnatural deformations (3) in the left wing). The colour scale of the changes (red, green and orange grid) highlights the anatomical changes of the upper part of the artefact. The process of crack propagation and deformation of the tip is also shown.

**Figure 24:**
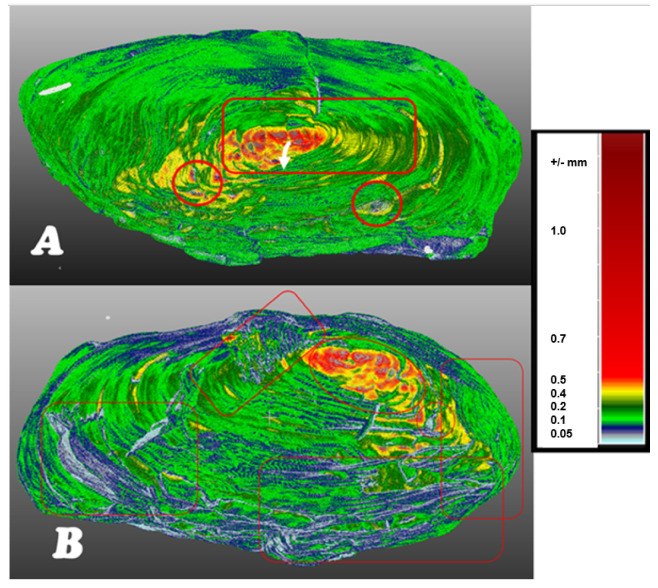
Deformation monitoring of the top of the artefact (comparison of the 2019 model with the 2018 reference model). (**A**) shows the dynamics of volumetric changes on the surface, and (**B**) shows the dynamics of changes in the anatomical structure of the artefact. The colour scale represents the annual process of shrinkage and deformation of the upper part of the artefact. The dynamics range from 0.1 mm (green) to 1.1 mm (red). The upper side of the artefact is mainly exposed to a more intensive deformation process. On the inside, smaller cracks, openings and pores can be seen. These touch the beginning of the crack, which extends over the entire length from the top to the handle area. Due to the ongoing deformation process in this part of the artefact, the annual deflection of the top of the Palaeolithic head in 2019 was 1.35 mm.

A comparison of the anatomical changes of the artefact after one year shows a tendency towards greater contraction of the tip (Figure 23 and Figure 24). This process also affects the condition of the longer crack running from the top towards the middle and to the plant part. The crack has widened (from 0.1 to 0.8 mm) and deepened further (from 0.1 to 1.4 mm) within a year. Deformation changes (widening of the cracks and smaller fractures—from 0.12 to 1.2 mm) were observed in the left wing of the artefact. The pronounced transverse fracture was stable over one year.

The deformation changes seen in Figure 24, which are not yet fully stabilised, indicate the hypothesis that the surface changes in the tip (bending, etc.) are mainly influenced by the crack that extends over the entire length in the upper part of the point. The dynamics of the changes at this crack have so far been faster than the dynamics of the changes at the lower fracture. A crack could be a major cause of bending of the plant part and the top of the point. In the long term, it could bring the risk of fracture or disintegration of the point.

### 6.6. Reconstruction of 3D Models of other Archaeological Objects and Composite Materials

**Figure 25:**
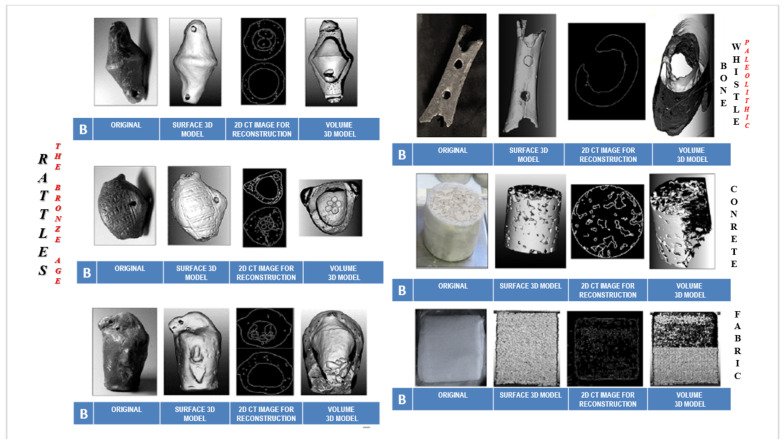
Six examples of reconstruction of 3D models from CT images of various archaeological objects and other composite materials. On the left are three clay rattles [93], one the right a Neanderthal bone whistle [69] (top), a cylindrical piece of concrete (middle) and some fabric (bottom).

Experimental tests of 3D model reconstruction with our algorithms were conducted on four smaller archaeological objects made of bone and clay and two other materials (concrete and fabric) to determine the robustness and quality of the algorithms for 3D model reconstruction of surfaces and volumes (Figure 25).

In all cases, the two algorithms have been shown to be suitable for the reconstruction of 3D models. As for the quality of the 3D models reconstructed and created with the algorithms *dAR3D* and *sAR3D*, they do not differ in any way from the quality of the models reconstructed with commercial software tools. It is significant that the segmentation algorithm (Figure 25) matches and even surpasses the quality, clarity and robustness of the 3D models reconstructed with commercial software tools. At the same time, the reconstructed models provide accurate volumetric data on the location and condition at the critical points.

## 7. Discussion

The iterative segmentation algorithm developed for reconstructing a 3D model from 2D micro-CT images of an archaeological object is only one of a number of current algorithms for processing 2D CT images or 3D CT sections. However, it is one of the first to use a computer vision approach to reconstruct 3D surface and anatomical models and addresses the weaknesses of traditional techniques for reconstructing 3D surface models from 2D images that occur when using conventional computer vision algorithms. It combines knowledge, methods and techniques from computed tomography and computer vision. It provides extremely accurate 3D surface and anatomical models that, together with high-quality CT images, can be a perfect copy of the original. Compared to other algorithms, it is simple and user-friendly. It is versatile and not specialised for narrow professional use (although it was developed for direct use by archaeologists and conservators). With reconstructed 3D models, it provides the user with a large amount of data and information and enriches archaeological documentation. The image data and information can be processed with already-available software tools for processing 3D network data. However, it is true that this makes the reconstruction of 3D models more expensive than traditional computer vision algorithms. With the financial availability of CT hardware, it is also becoming interesting for use in the heritage sciences. This is confirmed by the growing number of research projects and published professional contributions in all fields and not only in heritage studies.

With the developed segmentation algorithm we used in our specific archaeological case, the process of segmenting features in 2D images using the Roberts Edge detector, after reconstructing the 3D model (from any plane section) we obtain a depth image of the object in the case of a dynamic 3D visualisation or a printed 2.5-D image simulating the holographic method of recording 3D dimensional information about an archaeological object.

Our simplification of the preprocessing of 2D image files or 3D slices eliminates the need to use additional philtres (Rashidi, Vigorelli, Bakirov) for the segmentation process. It is also not necessary to use the translucent visualisation technique (Gaboutchian) of the 3D anatomical model. The depth view (a 2.5D depth view and not just a surface hologram of the outer edges of selected internal segmented features—see Figure 12, Figure 13, Figure 17, Figure 21, Figure 22, Figure 23) was not provided by any software tool for reconstructing 3D anatomical models from CT images. Anatomical views are therefore simulated in most cases by visualising only the outer edges of selected internal/anatomical deviations or features. To achieve a similar effect in visualising anatomical features—but without insight into the interior of the anatomical feature—a simple video sequence of 2D images of the reconstructed model in the selected plane is usually used to analyse the anatomical features of the 3D model. In this case, it is left to the user to visualise and localise the features and spatial properties of the selected anatomical feature by numerically recording metric data about its location, which is automatically acquired by the software tool. The volumetric correctness of the placement of the reconstructed 3D model in the Cartesian coordinate system may also be questionable. It may deviate from the original. Therefore, volumetric deviations and errors occur more frequently. This may complicate the volumetric analysis of the artefact or the design of conservation procedures. Especially when machine or deep learning, artificial intelligence or automatic or robotic processing methods or techniques are involved in the processes of analysis or conservation.

We do not exclude another way of visualising the anatomical structure of the object, but after reviewing the software tools of the user manual, such a possibility is neither described nor foreseen.

The highlighted features of the segmentation algorithm require critical consideration and implementation in new cases and in different practises to objectively assess its applicability. Our evaluation has shown that the new algorithms for reconstructing 3D models from 2D images CT have met expectations. Critical points in the internal structure of the Palaeolithic wooden point, which was the subject of our investigation, were successfully located. Anatomical features point to the causes of the surface changes. The need for further development of better conservation techniques for wet wood is highlighted.

In all cases, the algorithms qualitatively reconstructed the surface-based 3D models and the volumetric 3D models from CT images. The reconstructed 3D models do not differ from the quality of the models reconstructed or rendered using commercial software tools. This is an indication that the segmentation algorithm *sAR3D* reconstructs models that outperform those reconstructed with commercial software tools in terms of quality, clarity and volumetric accuracy.

The developed iterative algorithm for reconstructing 3D models from 2D microtomographic images of archaeological objects can be successfully used to create both surface-based and volumetric 3D models of X-ray scanned objects. They also enable the reconstruction of a three-dimensional 3D model after previously constraining the number of features specified by the user.

The algorithms were tested on seven practical examples and met expectations. The results answered the working questions (reconstruction of 3D models from 2D µCT images, internal deformation of the Palaeolithic artefact).

With the reconstructed 3D models, we were able to accurately identify, examine and document the internal structure of the artefact. Deformations (cracks, fractures, decay) are clearly visible and volumetrically localised. The 3D anatomical model successfully complemented the knowledge of the features and specificities of the alterations and revised the erroneous and not fact-based assessment of the alterations that was solely based on the surface-based 3D models.

The chosen hardware and software proved to be suitable to realise the set development and assessment goals of the study. It can form a basis for a possible standardisation of hardware and software for the reconstruction of computed tomography images into a 3D model.

The advantage of both algorithms is their ease of use. The user can reconstruct the 3D model according to his research objectives, with the appropriate hardware and open-source software configuration of the computer system, without any special mathematical or computer knowledge and without additional professional and technical support from a radiologist, computer scientist or physicist.

To implement the algorithm directly, the user needs:

- the selected number of CT images in the appropriate format (the set of images for reconstruction is not limited in number; in our case, for example, the set of CT images in the selected test objects ranged from 1100 to 3500 images);
- data and information from the radiologist about the thickness of the layer (d=t=z) and the type of X-ray beam;
- information about the possible inclination (in degrees) of the object mounted in the CT reader, and;
- information from the radiologist about the limits of the object (length – width – thickness) that can be detected by the selected CT reader with a single X-ray (scan).

The information and data provided are important for the accurate volumetric treatment of the object and its match to the original. Algorithms otherwise adapted for the use of computed tomography and 3D model reconstruction from CT images of small archaeological objects for archaeological documentation and for planning conservation and restoration procedures can also be robustly used in other cases (e.g., testing and analysis of materials or composites, in industrial control, etc.). The limit for the choice of the algorithm’s intended use depends on the technical possibilities of the chosen CT reader.

The algorithms are useful in the reconstruction of 3D models from 2D microtomographic or tomographic images acquired with all types of CT or µCT readers, as well as with other depth imaging readers (e.g., MRI, etc.). The segmentation algorithm can be fully adapted to the needs of different users and workspaces. It is simple and straightforward and does not require in-depth mathematical and computer knowledge of the direct user.

The quality of the reconstructed anatomical (3D volumetric) and surface-based 3D models from 2D µCT images is not significantly different from the quality of 3D models created with more expensive commercial software. The evaluation of the algorithms confirmed that the quality of the 3D model depends mainly on the quality of the input 2D CT or µCT images.

Given the amount of information contained in the internal texture of 2D µCT images, the *dAR3D* algorithm is significantly slower than the *sAR3D* in reconstructing the 3D model. The shorter reconstruction time of the 3D model with the *sAR3D* algorithm is due to the smaller amount of information in the segmented 2DµCT images. The difference between the two algorithms also lies in the size of the output file of the 3D model. The *dAR3D* file is significantly larger (Table 6).

It is critical to note that the current technological requirements for an optimal and rapid open-source implementation of the direct algorithm in the context of the use of personal computers for general use are not yet fully in place. With a large number of images (more than 1000) with high resolution (3000 × 3000 or more) and a high density of greyscale values of the HU or RGB matrix, the reconstruction process with a direct algorithm can be very time consuming. These circumstances significantly extend the time limits for effective reconstruction of 3D models, but they can be overcome with the help of cloud processing (4th data processing paradigm—op. Cit.) [94,95,96].

Advantages, disadvantages and limitations of both algorithms are summarised in Table 7.

The reconstruction of surface-based and volumetric 3D models of an archaeological object with both algorithms confirmed the extraordinary informative value of the model for an objective assessment of the current state of the artefact. The additional information about the condition of the artefact obtained in this way can also be of great help to conservators in selecting the most appropriate methods and techniques for conservation and protection.

## 8. Conclusions

Professional work in the field of cultural heritage conservation, archaeology, conservation and museology cannot be imagined without the use of new scientific methods and techniques originating in the natural sciences, or without the use of new computer and information technologies, artificial intelligence, collections of 3D models, multimedia, robotics, computer analysis of deformations and other modern technologies (Visual Languages, Remote Sensing, AI Neural Networks, Big Data, Data Science, etc.). Indeed, 3D surface-based models and volumetric 3D models (anatomical), volumetric 3D analyses and CT have become indispensable for documentation for the permanent, integrated conservation and presentation of tangible cultural heritage today and in the future.

The importance of 3D surface-based and volumetric 3D models and 3D computer visualisations enriches the standards for archaeological and cultural heritage recommended by the London Charter [10], the Seville Principles [11] and ratified international treaties.

At the same time, computed tomography and the reconstruction of 3D volumetric (anatomical) models and 3D surface-based models from tomographic or microtomographic images can effectively contribute to the implementation of Rule 4 of the Amendments to the UNESCO Convention for the Protection of the Underwater Cultural Heritage, which recommends that state authorities and institutions apply nondestructive methods to cultural heritage remains [6].

It would be appropriate for the archaeological and conservation professions to use noninvasive computed tomography more frequently than in the past and to model successful practises as standard methods and techniques for dealing with fragile and rapidly degradable cultural heritage remains.

Both the algorithms for reconstructing 3D surface-based and 3D volumetric models from CT images can be upgraded with new methods from the fields of computer vision, deep learning and artificial intelligence. By upgrading algorithms to reconstruct 3D models, we could complement or even replace the autonomous analytical function of humans in the treatment and evaluation of cultural heritage remains. Experiences with algorithms such as Deep Learning already represent a significant advance in the automatic recognition and processing of 2D images from computed tomography. Methods and techniques ranging from convolutional neural networks (CNNs) to variational auto-encoders (VAEs) are being used. These artificial intelligence methods are already delivering successful results in the automatic recognition of complex patterns in computed tomographic image data, particularly in the faster and more efficient segmentation of features and the automatic diagnosis of X-ray image conditions [97].

## Figures and Tables

**Table 1:** Comparison of volumetric values of 3D models of The Palaeolithic. wooden point (Deformation 2009/2013–2019). * — Manually measured and visually estimated. ** — Digitally measured with *CloudCompare* computer program. *** — Change (+ − µm) of volumetric value with CloudCompare by measurement before conservation procedure. **** — Change (+ − µm) of volumetric value with CloudCompare by measurement after the first phase of the conservation procedure (irrigation) with melamine resin ($C_{3}H_{6}N_{6}$). PP0—Palaeolithic wooden point. PP3D—Palaeolithic wooden point (3D surface-based model).

	In Situ	Ex Situ					
		3D Models					
3D Dimensions and Volume	PP0 2008	PP3D 2009	PP3D 2013	PP3D 2015	PP3D 2017	PP3D 2018	PP3D 2019
	0 *	1 **	2 **	3 **	4 **	5 **	6 **
	μM	μM	μM	μM	μM	μM	μM
*Lenght*	160,000	155,606	160,958	152,709	151,768	150,435	149,171
+/− % ***		100	+3.44	−1.86	−2.47	−3.32	−4.10
+/− % ****			100	−5.13	−5.71	−6.54	−7.32
*Width*	51,000	50,014	52,274	50,594	50,348	48,359	50,705
+/− % ***		100	+4.52	+1.16	+0.67	−3.31	+1.38
+/− % ****			100	−3.21	−3.68	−7.49	−3.00
*Thickness*	25,000	25,579	28,810	23,856	23,585	22,689	23,793
+/− % ***		100	+12.63	−6.74	−7.79	−11.30	−6.98
+/− % ****			100	−17.20	−18.14	−21.25	−17.41
*Volume*	μM${}^{3}$	μM${}^{3}$	μM${}^{3}$	μM${}^{3}$	μM${}^{3}$	μM${}^{3}$	μM${}^{3}$
		70,653.6	80,404.1	66,382.8	65,238.9	63,871.9	63,289.4
+/− % ***		100	+13.80	−6.04	−7.66	−9.60	−10.42
+/− % ****			100	−17.44	−18.86	−20.56	−21.29
			$C_{3}H_{6}N_{6}$				
			CONSERVATION				
			BEGIN	END			

**Table 2:** Volumetric changes of the artefact In situ and Ex situ (conservation with melamine resin). * In our case study there was an increase due to intensive irrigation of the artefact in an aqueous solution (the preparation phase for the conservation process). ** Confirmed using a CT scanner.

Protection		In Situ		Ex Situ		
**3D Model**		**PP-2009**	**PP-2013**	**PP-2015**	**PP-2017**	**PP-2018**
			Δ	Δ	Δ	Δ
Volumetric parameters +/−	Length	−	Enlargement + *	Reduction −	Reduction −	Reduction −
	Width	−	Enlargement + *	Reduction −	Reduction −	Reduction −
	Thickness	−	Enlargement + *	Reduction −	Reduction −	Reduction −
	Volume	−	Enlargement + *	Reduction −	Reduction −	Reduction −
	Deformation	−	No	Bending	Bending	
	Degradation	−	No	Crack	Crack	FE0000 Crack/Fracture /Shrinkage/Crumbs /Hole/ **
	Ovality	−	No	Change	Change	Change

**Table 3:** Code characteristics of the segmentation algorithm *sAR3D* for the reconstruction of 3D models from CT images.

	sAR3D Code Characteristics	
*Step*	*Code comment*	*Slide Master*
1	Preparation of the algorithm Defining, selecting and sorting image file names; scale; specify the name of the final file	imagefiles = dir(’*.tif’); n = natsortfiles((imagefiles.name)); nfiles = length(n); scale = 0.053; fid = fopen(’my.obj’,’wt’);
2	Loop (go through images by file name)	for ii =1:nfiles … end
3	Opens and reads each image file	currentimage = imread(currentfilename);
4	Obtaining and determining the x−y coordinate of points	Segment images and determine the coordinates of points from them
5	Add the third (*z*) coordinate to the points	z = repmat((ii * scale), [size(row,1) 1]); … points = [xy, z];
6	Write to specified end file (3D coordinate table)	result = cat(2,vert,string(points)); fprintf(fid, ’
7	Close the final 3D model file	Segment 3D model file

**Table 4:** Input data for the reconstruction of a 3D model from CT images.

Artefact	Number	Format	Image Size	Slice Thickness
Input Data	µCT Images			
Palaeolithic wooden point	2452 (year 2018) 2650 (year 2019)	TIFF	2699 × 2731 1012 × 1024	44.2 µm
Bone flute from Divje babe I	2649	TIFF	732 × 837	31.9 µm
Ceramic rattles	R1—1717 R2—1014 R3—1013	TIFF	1012 × 1024 1012 × 1024 1012 × 1024	51.9 µm 44.5 µm 62.7 µm
Different composites	B—1014 T—1014 (300)	TIFF	1012 × 1024	44.5 µm

**Table 5:** Output data of the reconstructed 3D model from CT images.

Artefact	3D Model—File Size		
	dAR3D	Format	sAR3D
Palaeolithic wooden point	8.18 GB (year 2018) 7.7 GB (year 2019)	OBJ	196 MB (year 2018) 193 MB (year 2019)
Bone flute from Divje babe I	4.68 GB	OBJ	166 MB
Ceramic rattles	R1—5.12 GB R2—3.26 GB R3—2.49 GB	OBJ	R1—132.0 MB R2—89.2 MB R3—68.1 MB
Different composites	B—14.9 GB	OBJ	B—280 MB
	T—1.8 GB	OBJ	T—69 MB

**Table 6:** Reconstruction time and output file size of algorithms *dAR3D* and *sAR3D*.

Artefacts	3D Model—Reconstruction Time and Output File Size				
	**dAR3D**	**File Size**	**Format**	**sAR3D**	**File Size**
**Palaeolithic wooden point**	48 h (year 2018) 36 h (year 2019)	8.18 GB 7.7 GB	OBJ	1.10 h (year 2018) 1.04 h (year 2019)	196 MB 193 MB
**Bone flute from** **Divje Babe I**	24 h	4.68 GB	OBJ	55’	166 MB
**Ceramic rattles**	R1—23.9 h R2—16.0 h R3—11.7 h	5.12 GB 3.26 GB 2.49 GB	OBJ	R1—45’ R2—30’ R3—24’	132.0 MB 89.2 MB 68.1 MB
**Different composites**	CONCRETE—18.1 h FABRIC—8.7 h	14.9 GB 1.8 GB	OBJ	CONCRETE—78’ FABRIC—20’	280 MB 69 MB

**Table 7:** Advantages, limitations and deficiencies of algorithms *dAR3D* and *sAR3D*.

	*dAR3D*	*sAR3D*
Advantages	- Complete surface and volume reconstruction of the 3D model; suitable for quality and complete addition of the original. - Suitable for the reconstruction of small artefacts. - Suitable for the reconstruction of up to 300 CT, MRI, ultrasound, MMG... 2D images.	- Fast, reliable and efficient reconstruction of the 3D model. - High-quality and accurate surface 3D model for visualisation and addition. - High-quality and more segmented 3D volume model according to selected characteristics. - More vivid and selective presentation and analysis of 3D model data. - Adaptation to the interests and needs of the end user. - Simple and easy by the end user. - Robustness (can be used in various fields). - Suitable for processing and processing a large number of CT, µCT, then- CT, …, MRI, ultrasound, MMG, …2D images (1000 <n). - Ability to remove unwanted data. - Lower memory and hardware load. - Efficient and fast operation regardless of the number of 2D images reconstructed. - Fast and efficient comparison and processing of volumetric data from surface and volume 3D model. - Efficient and fast implementation of deformation monitoring. - Smaller and more suitable file of reconstructed 3D models for further processing.
Limitations and Deficiencies	- Longer time intervals of 3D model reconstruction from µCT images (t = 25–50 x; depending on architecture and hardware capabilities). - Optimal processing in the range of up to 300 2D images. - Extremely large files when reconstructing from a larger set of 2D images (over 1000) with a higher resolution (e.g., 15–50 GB). - Increased saturation and therefore the risk of noise and poorer contrast. - Poorer quality (contrast...) due to a larger set of greyscales HU or RGB scales. - Lots of useless and unstructured data.	

## Abbreviations

The following abbreviations are used in this manuscript:

CT	computed tomography
dAR3D	direct algorithm for 3D model reconstruction
sAR3D	segmentation algorithm for 3D model reconstruction
